# Type I Interferons Ameliorate Zinc Intoxication of *Candida glabrata* by Macrophages and Promote Fungal Immune Evasion

**DOI:** 10.1016/j.isci.2020.101121

**Published:** 2020-05-04

**Authors:** Michael Riedelberger, Philipp Penninger, Michael Tscherner, Bernhard Hadriga, Carina Brunnhofer, Sabrina Jenull, Anton Stoiber, Christelle Bourgeois, Andriy Petryshyn, Walter Glaser, Andreas Limbeck, Michael A. Lynes, Gernot Schabbauer, Guenter Weiss, Karl Kuchler

**Affiliations:** 1 Medical University of Vienna, Center for Medical Biochemistry, Max Perutz Labs Vienna, Campus Vienna Biocenter, Vienna, Austria; 2 Institute of Chemical Technologies and Analytics, TU Wien, Vienna, Austria; 3 Department of Molecular and Cell Biology, University of Connecticut, CT, USA; 4 Institute for Vascular Biology and Thrombosis Research, Center for Physiology and Pharmacology, Medical University of Vienna, Vienna, Austria; 5 Department of Internal Medicine II, Infectious Diseases, Immunology, Rheumatology, and Pneumology, Medical University of Innsbruck, Innsbruck, Austria; 6 Christian Doppler Laboratory for Arginine Metabolism in Rheumatoid Arthritis and Multiple Sclerosis, Vienna, Austria

**Keywords:** Immunology, Immune Respons, Immune Response

## Abstract

Host and fungal pathogens compete for metal ion acquisition during infectious processes, but molecular mechanisms remain largely unknown. Here, we show that type I interferons (IFNs-I) dysregulate zinc homeostasis in macrophages, which employ metallothionein-mediated zinc intoxication of pathogens as fungicidal response. However, *Candida glabrata* can escape immune surveillance by sequestering zinc into vacuoles. Interestingly, zinc-loading is inhibited by IFNs-I, because a Janus kinase 1 (JAK1)-dependent suppression of zinc homeostasis affects zinc distribution in macrophages as well as generation of reactive oxygen species (ROS). In addition, systemic fungal infections elicit IFN-I responses that suppress splenic zinc homeostasis, thereby altering macrophage zinc pools that otherwise exert fungicidal actions. Thus, IFN-I signaling inadvertently increases fungal fitness both *in vitro* and *in vivo* during fungal infections. Our data reveal an as yet unrecognized role for zinc intoxication in antifungal immunity and suggest that interfering with host zinc homeostasis may offer therapeutic options to treat invasive fungal infections.

## Highlights

• Metallothioneins shuttle zinc into macrophage phagosomes to elicit pathogen killing
• Zinc sequestration by metallothioneins drives potent fungicidal ROS responses
• IFN-I signaling dysregulates host zinc homeostasis during invasive candidiasis
• IFNs-I suppress zinc intoxication and promote pathogen fitness and immune evasion

## Graphical Abstract

**Figure:**
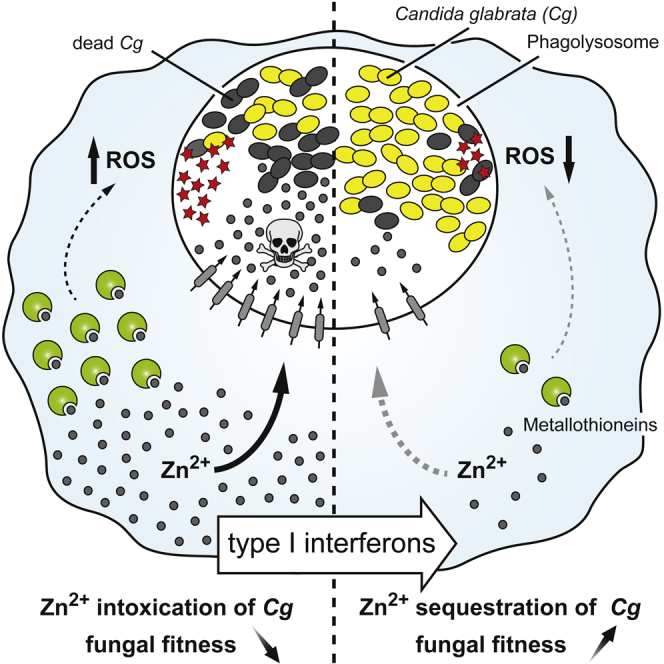

## Introduction

*Candida glabrata* represents an opportunistic intracellular human fungal pathogen, causing life-threatening infections in immunocompromised patients (Pappas et al., 2018). Of note, *C. glabrata* (*Cg*) infections have been sharply increasing over the past two decades, yet classical therapeutic options are limited owing to the inherent resistance against echinocandins and azoles (Fisher et al., 2018, Perlin et al., 2017, Taff et al., 2013, Vale-Silva and Sanglard, 2015). In addition, adaptive evolution equipped this fungal pathogen with a vast repertoire of defense mechanisms that facilitate immune evasion (Kasper et al., 2015, Kumar et al., 2019). For example, after phagocytosis by myeloid immune cells, *Cg* establishes an environmental niche in the host enabling growth and proliferation inside innate immune cells by suppressing the generation of reactive oxygen species (ROS), inhibiting phagolysosomal maturation, and nutrient acquisition through several pathways (Kumar et al., 2019, Seider et al., 2011). The immune defense in turn mounts local and systemic pro-inflammatory responses to boost clearing of *Cg* by macrophages and neutrophils (Netea et al., 2015).

The well-known type I interferon (IFN-I) cytokine family has been implicated in most if not all microbial and viral infections (McNab et al., 2015, Stifter and Feng, 2015). Remarkably, IFNs-I set pro-inflammatory stimuli aimed at supporting immune surveillance and defense. However, pro-inflammatory IFN-I actions can be both beneficial and detrimental in infectious settings, particularly in cases where excessive immunopathology drives self-imposed “collateral” oxidative damage to host tissues (Majer et al., 2012, McNab et al., 2015). Indeed, we have previously reported that IFNs-I drive the persistence of *Cg* in brain, liver, and spleen of *Ifnar1*^*−/−*^ mice, thereby dysregulating the cellular iron homeostasis in macrophage subsets, which inadvertently facilitates fungal iron acquisition that enhances fungal fitness (Bourgeois et al., 2011, Riedelberger et al., 2020).

Among many pathways, iron homeostasis regulation appears as a major target of IFN-I and -II signaling (Nairz et al., 2008, Nairz et al., 2018, Riedelberger et al., 2020). In addition, interferons share a common role in the regulation of zinc (Zn) homeostasis. For example, IFN-I responses reduce plasma Zn concentrations by inducing hepatic metallothionein expression in various model organisms (Guevara-Ortiz et al., 2005, Van Miert et al., 1990, Morris and Huang, 1987, Sato et al., 1996), as well as in human cells (Friedman and Stark, 1985, Nagamine et al., 2005, Read et al., 2017). IFN-γ regulates plasma Zn concentrations (Morimoto et al., 1987), Zn transporter expression in intestinal epithelial cells and pancreatic β-cells (Egefjord et al., 2009, Melia et al., 2019), as well as Zn levels in mycobacteria-containing vacuoles (Wagner et al., 2005a). In contrast, IFN-λ3 increases intracellular Zn levels (Read et al., 2017). Although accumulating evidence suggests an interferon/Zn axis, the molecular players controlling the dynamic response have remained enigmatic. Interestingly, the interferon/Zn axis is under reciprocal control, because interferons regulate both cellular and systemic zinc levels. Labile Zn is required for optimal STAT1-dependent IFN signaling (Reiber et al., 2017) and modulates TLR signaling as well (Brieger et al., 2013, Maares and Haase, 2016, Wessels et al., 2017).

Proper innate and adaptive immune responses require tightly regulated intracellular Zn levels (Weiss and Carver, 2018). Indeed, Zn deficiency arising from malnutrition or mutations increases the susceptibility for infections and various other diseases (Ferreira and Gah, 2017, Lopez and Skaar, 2018, Vaeth and Feske, 2018). Of note, invading microbial pathogens inevitably rely on Zn for successful host infection and propagation within the host owing to metabolic restrictions within a given host. For example, neutrophils use calprotectin secretion to sequester Zn, thus creating a Zn-limited environment for pathogens (Zackular et al., 2015). By contrast, macrophages exert at least two context-dependent Zn defense strategies for several microbes (Subramanian Vignesh and Deepe, 2016). After phagocytosis of *Histoplasma capsulatum*, macrophages shuttle Zn from the phagolysosome into the cytoplasm to elicit fungal Zn starvation and elevated ROS production (Subramanian Vignesh et al., 2013a, Subramanian Vignesh et al., 2016). In contrast, during infections with *Mycobacterium tuberculosis*, *Escherichia coli*, and *Salmonella enterica serovar Typhimurium* (*S. Typhimurium*), macrophages actually trigger phagolysosomal Zn accumulation to drive Zn intoxication and killing of the invading bacterial pathogens (Botella et al., 2011, Kapetanovic et al., 2016, Stocks et al., 2019). Thereby, macrophages exploit Zn-binding metallothioneins to control intracellular Zn sequestration and spatiotemporal Zn distribution during antimicrobial responses (Subramanian Vignesh and Deepe, 2017).

Here, we show that IFN-I responses dysregulate the Zn homeostasis in macrophages *in vitro* and *in vivo* during systemic *Cg* infections. We provide an in-depth mechanistic view of how macrophages employ Zn intoxication of fungal pathogens as a fungicidal defense. However, IFN-I signaling attenuates this defense by transcriptional suppression of host Zn transport systems. This response inadvertently leads to altered spatiotemporal Zn distribution that impairs otherwise fungicidal ROS production in macrophages. Invasive fungal infections by *Cg* elicit strong IFN-I responses, which in turn dysregulate splenic Zn homeostasis and the antifungal response of splenic macrophages. Thus, targeting the IFN-I-driven host immune surveillance or targeting fungal Zn homeostasis might provide therapeutic concepts to treat disseminated fungal infections or other microbial pathogens in general. Such therapeutic concepts targeting nutritional immunity would be highly advantageous, because they may help combating *Cg* or *C. auris*, as these fungal pathogens display pronounced inherent antifungal drug resistance rendering them refractory to conventional treatment.

## Results

### IFNs-I Dysregulate Zn Homeostasis Genes in the Spleen during Systemic *Cg* Infections

We have previously reported that IFNs-I are detrimental for the host during systemic *Cg* infections (Bourgeois et al., 2011). *Ifnar1*^*−/−*^ mice (which lack the Interferon alpha and beta receptor subunit 1) are unable to respond to IFN-I signals (Müller et al., 1994), and exhibit reduced fungal loads in liver, kidney, and spleen (Bourgeois et al., 2011). Because the spleen is a key target for microbial pathogens disseminating through the blood (Borges da Silva et al., 2015a, Borges da Silva et al., 2015b), we investigated the effects of IFN-I signaling on the splenic response during fungal infections. Therefore, wild-type (WT) and *Ifnar1*^*−/−*^ mice were intravenously infected with 5 x 10^7^*Cg* colony-forming units (CFUs) per 25 g mouse weight. After 1, 3, 7, and 14 days post-infection, spleens were harvested and transcriptional profiling of splenic responses was performed by microarrays (Table S1). We showed that IFNs-I dramatically dysregulate iron homeostasis in macrophage populations and the spleen. Thereby, *Cg* inadvertently obtains IFN-I-mediated access to phagolysosomal iron pools, which facilitates fungal replication and persistence (Riedelberger et al., 2020).

**Figure 1:**
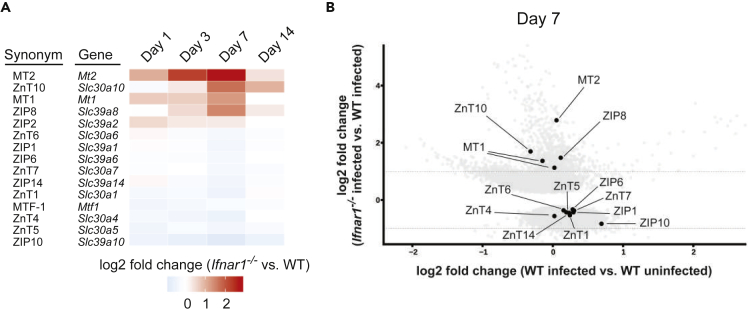
IFN-I Signaling Dysregulates the Splenic Transcriptional Response of a Zn-Homeostasis-Related Gene Set during Invasive *C. glabrata* Infection (A) Microarray analysis of DEGs in WT and *Ifnar1*^*−/−*^ spleens after intravenous *Cg* infection for 1, 3, 7, or 14 days (n = 3 mice per group). Total RNA extracted from spleens was hybridized to the SurePrint G3 Mouse GE 8 × 60K Microarray chip (Agilent Technologies). Heatmap shows the differentially expressed Zn-homeostasis-related genes in infected *Ifnar1*^*−/−*^ mice relative to infected WT mice after normalization to uninfected mice (cut-off: FDR<0.05). All listed genes were up- or downregulated at least at one time point during the course of systemic infection (p < 0.05). (B) Scatter plot of DEGs from WT and *Ifnar1*^*−/−*^ spleens at day 7 of systemic *Cg* infection. Each dot represents one probe on the microarray, and black dots (FDR <0.05) correspond to Zn homeostasis-related genes. DEG, differentially expressed gene; FDR, false discovery rate; see also Figure S1.

Of note, many pathogenic infections can cause subtle alterations of metal availabilities in different cellular compartments of macrophages (Wagner et al., 2005a, Wagner et al., 2005b). Further, alterations of zinc homeostasis as well as interferon signaling impact the hepatic immune responses in different disease models (Read et al., 2017). Thus, based on the effects of IFNs-I on splenic iron homeostasis regulation (Riedelberger et al., 2020), we assumed a possible involvement of IFN-I signaling in Zn homeostasis during *Cg* infections. Based on an in-depth literature search, we used a defined set of immunity-associated Zn homeostasis genes for the bioinformatic analysis of our existing microarray dataset (Table S2) (Riedelberger et al., 2020). Interestingly, *Cg*-infected *Ifnar1*^*−/−*^ spleens exhibited dysregulated expression of genes involved in the regulation of Zn homeostasis at least once during the time course of systemic infection (Figure 1A). For example, Zn importers (ZIPs; *Slc39a1-14*), Zn exporters (ZnTs; *Slc30a1-10*), metallothioneins (MT), as well as the transcriptional master regulator for Zn homeostasis MTF-1 showed significant expression changes upon loss of IFN-I signaling. Strikingly, the metallothioneins MT1 and MT2, which are cytoplasmic Zn chaperones orchestrating potent antimicrobial defences (Subramanian Vignesh and Deepe, 2017), were swiftly induced upon *Cg* infection (Figures S1A and S1B) and showed highest expression in *Ifnar1*^*−/−*^ spleens at day 7 after fungal challenge (Figure 1B). Thus, these data demonstrate that IFN-I signaling in response to *Cg* infections alters the splenic response with respect to Zn homeostasis.

### IFNs-I Alter Zn Transporter Expression upon *Cg* Infection

**Figure 2:**
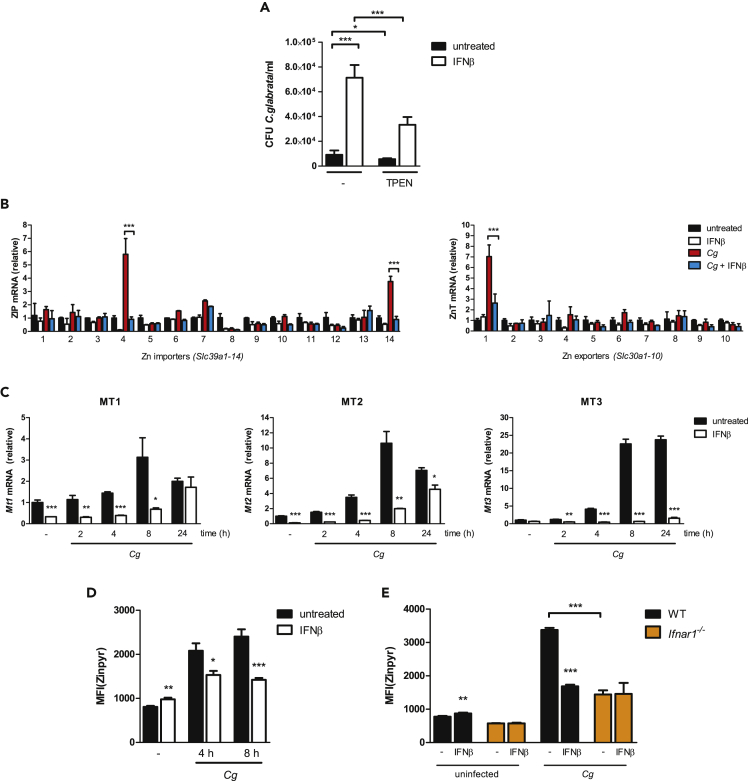
IFNs-I Inhibit Zn Transporter Expression and Prevent Zn Burst in *C. glabrata*-Infected BMDMs (A) *In vitro* survival assay of *Cg* after 24-h interaction with WT BMDMs untreated or IFNβ-treated upon pre-incubation with TPEN for 1 h. (B) RT-qPCR analysis of *Slc39a1-14* and *Slc30a1-10* mRNA levels in WT BMDMs untreated or IFNβ-treated during *Cg* infection (normalization to *Actb*). One-way ANOVA with Bonferroni's post hoc analysis. (C) RT-qPCR analysis of *Mt1*, *Mt2*, and *Mt3* mRNA levels in WT BMDMs untreated or IFNβ-treated during *Cg* infection (normalization to *Actb*). (D) Zinpyr assay of WT BMDMs untreated or IFNβ-treated after 4 and 8 h of *Cg* infection. (E) Zinpyr assay of BMDMs untreated or IFNβ-treated during *Cg* infection for 8 h. Data are representative of two (B–E) or three (A) independent experiments. Mean and SD are shown; ∗ p value < 0.05, ∗∗ p value < 0.01, ∗∗∗ p value < 0.001; (A and C–E) Student's t test; (B) one-way ANOVA with Bonferroni's post hoc analysis. See also Figure S2.

Macrophages utilize several sophisticated mechanisms to ensure proper Zn homeostasis regulation during microbial infections (Subramanian Vignesh and Deepe, 2016). To investigate in detail how IFNs-I modulate Zn homeostasis in antifungal immunity, we infected primary bone-marrow-derived macrophages (BMDMs) with *Cg*. Interestingly, overnight treatment of BMDMs with IFNβ subsequently increased fungal survival upon macrophage infection (Figure 2A). When BMDMs were pre-treated with the cell-permeable, high-affinity Zn chelator TPEN before *Cg* challenge, the IFNβ-mediated effects on fungal survival were partially rescued (Figure 2A). BMDM functions such as *Cg* phagocytosis, autophagy, as well as cellular viability remained unaltered by IFNβ. However, IFNs-I increase the intracellular replication of phagocytosed *Cg* after 4 h of BMDM infection (Riedelberger et al., 2020). Thus, IFNs-I promote fungal survival in macrophages, which partly depends on altered Zn homeostasis regulation.

Next, we performed an unbiased expression analysis of Zn importers (ZIPs; *Slc39a1-14*) and Zn exporters (ZnTs; *Slc30a1-10*). Upon BMDM infection with *Cg*, ZIP4 and ZIP14, as well as ZnT1, were highly upregulated, but were strikingly suppressed by IFNα and IFNβ treatment (Figures 2B and S2A). Although ZIPs are responsible for Zn mobilization into the cytoplasm (Subramanian Vignesh and Deepe, 2016), ZnT1 may mediate Zn transport into mycobacteria-containing phagolysosomes for Zn intoxication (Botella et al., 2011). In addition, IFNα and IFNβ inhibited the *Cg*-induced expression of the metallothioneins *Mt1*, *Mt2*, and *Mt3* (Figures 2C and S2A). These data suggest that *Cg* infections induce Zn mobilization and, perhaps Zn transport to intracellular organelles in macrophages, which is suppressed by IFN-I responses.

The IFN-I-mediated dysregulation of Zn homeostasis genes prompted our hypothesis that IFNs-I might alter Zn metal ion concentrations in BMDMs upon *Cg* infections. Thus, we performed inductively coupled plasma mass spectrometry (ICP-MS) to quantify Zn concentrations in whole-cell lysates of *Cg*-infected BMDMs after excluding fungal debris. Although Zn levels remained unaltered upon IFNβ treatment (Figure S2B), the quantification of total steady-state Zn concentrations might miss dynamic exchanges between Zn storage organelles (e.g. mitochondria, ER, Golgi) with the labile, bioactive Zn pool in the cytoplasm (Kambe et al., 2015). To quantify cytoplasmic Zn alterations, we stained BMDMs with the Zn-specific fluorescent dye Zinpyr (Walkup et al., 2000), which binds intracellular, labile Zn^2+^ ions, leading to increased Zinpyr fluorescence (Figueroa et al., 2015). Thus, intracellular Zn levels positively correlate with the fluorescence intensity of Zinpyr. As observed by flow cytometry analysis, overnight treatment with IFNβ resulted in slightly increased Zinpyr fluorescence and, therefore, elevated intracellular Zn levels in uninfected BMDMs (Figure 2D). Strikingly, after 4 h of *Cg* infection, a burst of free Zn was observed in BMDMs, which was diminished by IFNβ treatment (Figures 2D and S2C). However, the IFNα/β-mediated inhibition of the Zn burst was absent in *Ifnar1*^*−/−*^ BMDMs (Figures 2E and S2D). Of note, the basal, constitutive IFN-I signaling (Gough et al., 2012) in BMDMs was still required for the subsequent Zn burst following *Cg* infections, because *Ifnar1*^*−/−*^ BMDMs failed to upregulate intracellular Zn levels (Figures 2E and S2D). Taken together, these results show that IFNs-I suppress ZIP/ZnT gene expression in infected macrophages, thus preventing the burst of free intracellular Zn in BMDMs. By contrast, basal IFNAR1 signaling is still required for Zn mobilization during fungal challenge, which reflects a paradoxical dichotomy IFNs-I can exhibit in certain infection settings.

### Zn Shuttling to *Cg*-Containing Phagolysosomes Is Attenuated by IFNs-I

**Figure 3:**
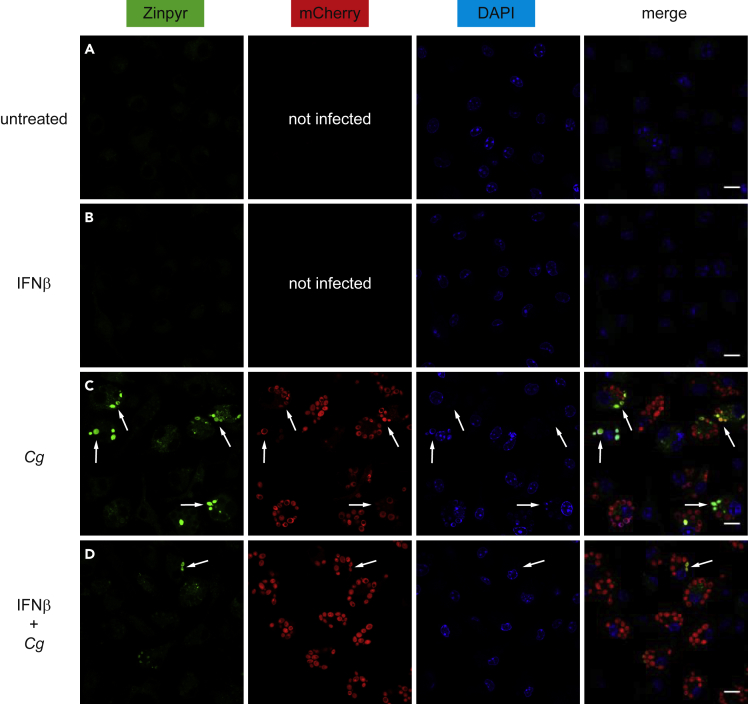
IFNs-I Prevent Zn Transport into *C. glabrata*-Containing Phagolysosomes (A–D) Confocal microscopy analysis of Zn (Zinpyr; green), mCherry-expressing *Cg* (red), and nucleus (DAPI; blue) in untreated or IFNβ-treated WT BMDMs after 4 h of *Cg* infection. Merge, overlay of all three channels. Arrows point at Zn^high^ yeast cells within infected BMDMs. The scale bar represents 10 μm. Data are representative of two (A–D) independent experiments. See also Figure S3.

**Figure 4:**
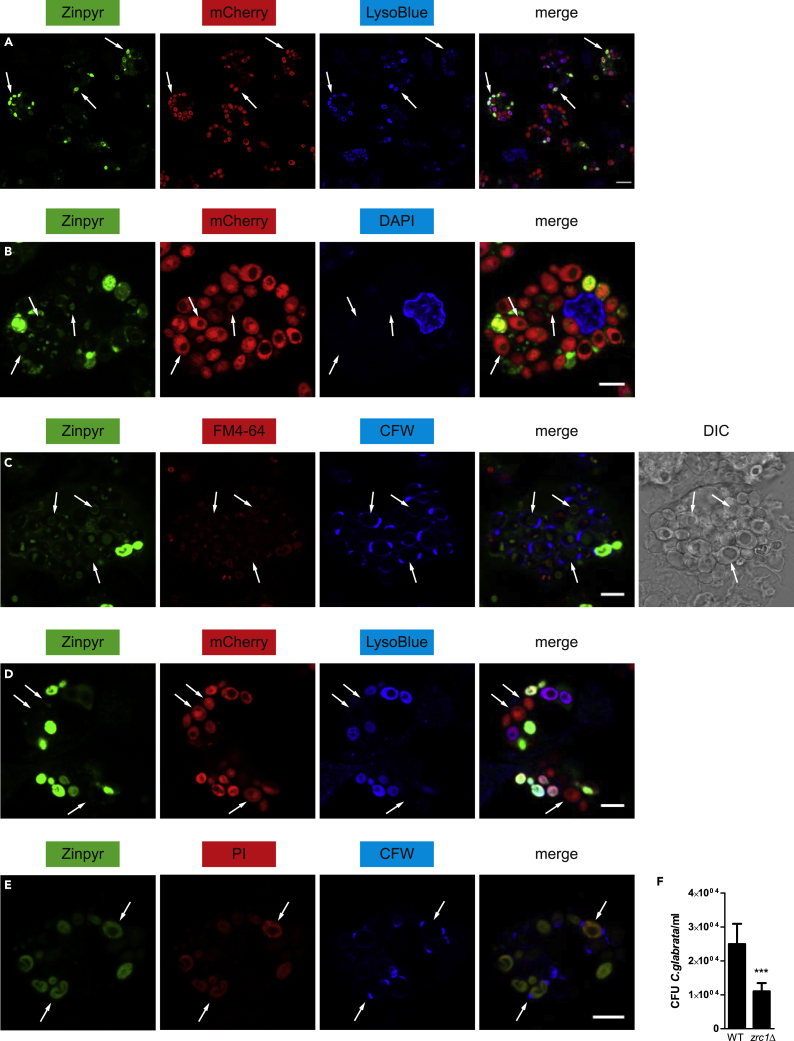
*C. glabrata* Counteracts Phagolysosomal Zn Stress by Vacuolar Zn Sequestration (A–E) Confocal microscopy analysis in WT BMDMs after 4 h of *Cg* infection. Analysis of Zn (Zinpyr; green), mCherry-expressing *Cg* (red), vacuolar membranes of *Cg* (FM4-64; red), dead fungal cells (PI; red), acidic/lysosomal staining (LysoBlue; blue), nucleus (DAPI; blue), and Calcofluor-White-stained *Cg* (CFW; blue). Arrows point at (A) yeasts within phagolysosomes, (B) Zn^low^*Cg*(C) vacuolar localization of Zn in *Cg*(D) Zn^low^*Cg* prevents phagolysosomal maturation, and (E) Zinpyr^+^ PI^+^ fungal cells. Merge, overlay of all three channels. DIC, differential interference contrast. The scale bar represents (A) 10 μm or (B–E) 5 μm. (F) *In vitro* survival assay of WT and zrc1Δ Cg after 24-h interaction with WT BMDMs. Data are representative of two (A–E) or four (F) independent experiments. Mean and SD are shown; ∗∗∗ p value < 0.001 (Student's t test). See also Figure S3.

Next, we wanted to visualize the spatiotemporal regulation of Zn distribution in *Cg*-infected BMDMs using confocal microscopy and Zinpyr staining. Uninfected BMDMs showed minor Zinpyr fluorescence within the cytoplasm (Figures 3A and 3B). Strikingly, upon infection with a mCherry-expressing *Cg* strain, we identified two discrete BMDM populations. First, Zn-resting BMDMs showed only minor cytoplasmic Zn signals and second, a Zn-activating BMDM population, which triggered a cytoplasmic Zn burst and Zn accumulation in phagocytosed *Cg* yeast cells, as judged from the observed Zinpyr-mCherry colocalization (Figures 3C, S3A, and S3B). Indeed, Zn^high^*Cg* cells colocalized with acidic phagocytic compartments, as evident from the staining by LysoBlue (Figure 4A), showing that Zn is transported into *Cg*-engulfing mature phagolysosomes. However, in IFNβ-treated BMDMs, Zn^high^*Cg* are barely detected, suggesting that IFNβ indeed inhibits phagolysosomal Zn accumulation (Figure 3D).

Interestingly, two discrete populations of *Cg* were visualized within macrophages, which were the aforementioned Zn^high^*Cg* population and a Zn^low^ population (Figure 4B). Although Zn^high^ yeast cells were entirely stained by Zinpyr, only an intracellular organelle was Zinpyr-positive in Zn^low^ fungal cells. Of note, the vacuole in the non-pathogenic yeast *Saccharomyces cerevisiae* acts as crucial detoxification system upon Zn stress, because large amounts of Zn can be transported and stored in this organelle (Gerwien et al., 2018, Simm et al., 2007). Indeed, by using the vacuolar membrane-specific dye FM4-64, we identified the vacuole as the Zn-rich organelle within Zn^low^*Cg* (Figures 4C and S3C). In addition, Zn^low^*Cg* remained negative for lysosomal staining, showing that Zn^low^*Cg* were able to actively inhibit phagolysosomal maturation (Figure 4D). In contrast, Zn^high^*Cg* failed to suppress lysosomal fusion and, therefore, localized only to acidic phagolysosomes. Notably, Zn detoxification via vacuolar sequestration and inhibition of phagolysosomal maturation by *Cg* are active processes that require a functional fungal metabolism (Kumar et al., 2019). Thus, we speculated that the Zn^low^*Cg* population contains viable yeast cells within macrophages, whereas Zn^high^*Cg* cells were killed by macrophages. Indeed, Zn^high^*Cg* corresponded to dead fungal cells owing to their positive PI staining, whereas Zn^low^*Cg* remained viable because they were PI-negative (Figure 4E). Of note, the regulation of Zn homeostasis in *Cg* in general is little understood. However, in *S. cerevisiae,* Zn sequestration into the vacuole is regulated by Zrc1 and Cot1 (MacDiarmid et al., 2000, Wilson and Deepe, 2019). Indeed, a genetic deletion of *ZRC1* in *Cg* resulted in a substantial survival defect upon BMDM infection (Figure 4F), showing that *ZRC1* is crucial for fungal immune evasion by *Cg*.

Taken together, these results show that upon *Cg* infection, BMDMs induce a Zn burst for subsequent Zn transport and accumulation into *Cg*-containing phagolysosomes. Thereby, *Cg* cells fail to suppress lysosomal fusion, leading to Zn accumulation and fungal killing, whereas viable, metabolically active Zn^low^*Cg* manage to inhibit phagolysosomal maturation and detoxify Zn by sequestration into the fungal vacuole. However, IFNs-I suppress the spatiotemporal Zn distribution in BMDMs, implying a survival and fitness advantage for *Cg*.

### IFNs-I Suppress Zn Intoxication of *Cg*

Next, we wanted to investigate in detail the relationship between Zn concentrations and fungal survival during host-pathogen interactions. When we performed flow cytometry analysis, we again observed Zn-resting and Zn-activating macrophage populations after *Cg* infections, which can be discriminated by their different Zinpyr fluorescence intensity (Figure S4A). Upon *Cg* infection, Zn levels remained similar in Zn-resting BMDMs when compared with uninfected BMDMs. By contrast, Zn levels where increased in *Cg*-infected, Zn-activating BMDMs. Interestingly, IFNβ treatment reduced the generation of Zn-activating BMDMs during *Cg* infection (Figure S4A). Thus, we reasoned that the appearance of Zn-activating BMDMs might represent an antifungal defense mechanism, which macrophages use for Zn intoxication and killing of *Cg*. Indeed, high Zn concentrations were toxic for *Cg* and prevented fungal growth (Figure S4B), which is fully consistent with previous reports (Crawford et al., 2018, Gerwien et al., 2018).

**Figure 5:**
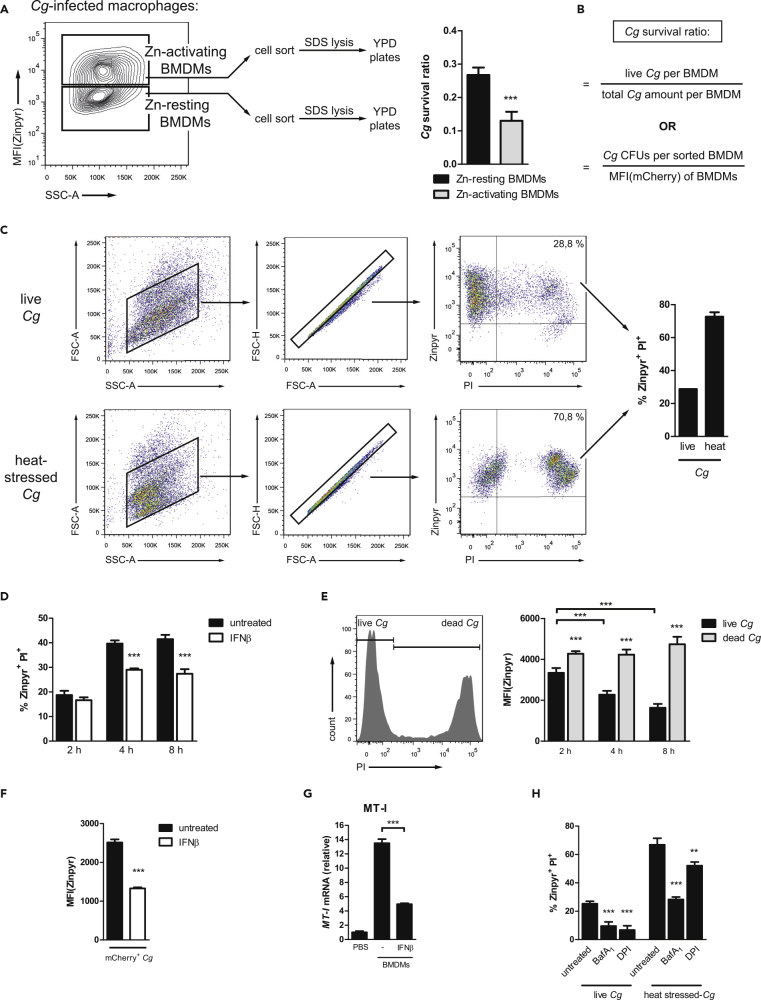
IFNs-I Inhibit Fungal Zn Intoxication during BMDM Infection (A) Schematic representation and survival ratio quantification of *Cg* isolated from FACS-sorted Zn-resting and Zn-activating WT BMDMs after 4 h of infection. (B) Calculation of the *Cg* survival ratio. The amount of CFUs per BMDM was normalized to the MFI(mCherry) of the respective BMDM population. (C) Fungal Zn intoxication assay. Gating strategy and quantification of Zinpyr^+^ PI^+^*Cg* after WT BMDM infection with live or heat-stressed fungal cells for 2 h. (D) Fungal Zn intoxication assay of *Cg* isolated from untreated or IFNβ-treated WT BMDMs. (E) Histogram and quantified Zinpyr fluorescence intensity of live and dead Zinpyr^+^*Cg* isolated from WT BMDMs after several hours of infection. (F) Zinpyr-based fungal Zn acquisition of mCherry^+^*Cg* isolated from WT BMDMs after 8 h of infection. (G) RT-qPCR analysis of *MT-I* mRNA levels in *Cg* upon infection of untreated or IFNβ-treated BMDMs for 4 h (normalization to *ACT1*). (H) Fungal Zn intoxication assay of live or heat-stressed *Cg* isolated from BMDMs untreated or treated with Bafilomycin A_1_ or DPI after 2 h of fungal challenge. Data are representative of two (A, C, G, and H) or three (D–F) independent experiments. Mean and SD are shown; ∗∗∗ p value < 0.001 (Student's t test). See also Figures S4 and S5.

In order to test our hypothesis, we conducted two approaches. First, after mCherry^+^*Cg* infection, we separated Zn-resting and Zn-activating BMDMs via cell sorting. Then, following BMDM lysis, we plated the cell lysates on YPD plates to quantify surviving fungal CFUs (Figure 5A). Thereby, we determined the *Cg* survival ratio in BMDMs (Figure 5B), which is calculated as the surviving *Cg* CFUs per sorted BMDMs divided by the total amount of *Cg* per sorted BMDM (represented by total mCherry fluorescence). Strikingly, *Cg* viability was strongly reduced in Zn-activating BMDMs when compared with Zn-resting BMDMs (Figure 5A), showing that Zn-activating BMDMs can execute efficient fungal killing. A graphic illustration depicts this notion (Figure S4C). Second, we aimed to quantify the inviable Zn^high^ *Cg* population upon host-pathogen interactions. Therefore, we infected BMDMs with WT *Cg* and incubated BMDMs with Zinpyr at the end of infection (to stain Zn^high^ yeast cells). We then lysed BMDMs to release intracellular *Cg* and finally stained these fungal cells with PI. Flow cytometry allows for easy separation of fungal cells from smaller particles using forward scatter/side scatter (FSC/SSC) discrimination (Figures 5C and S5A). Interestingly, after 2 h of infection, almost all *Cg* cells isolated from BMDMs were Zinpyr-positive, with about 30% of these Zinpyr^+^ *Cg* being inviable since also PI-positive (Figure 5C). As expected, the Zinpyr^+^ PI^+^ *Cg* population further increased, when alive but heat-stressed fungal cells (1 min at 65°C) were used as a positive control for BMDM infection. Next, we performed a time course experiment to follow the Zinpyr^+^ PI^+^ *Cg* population over time. We observed that *Cg* was rapidly loaded with Zn, and after 4 h, approximately 40% of all fungal cells were within the Zn^high^ PI^+^ dead *Cg* population (Figure 5D). When we discriminated between live (PI-negative) and dead (PI-positive) *Cg*, we observed that Zn levels for dead *Cg* remained high throughout the infection course (Figure 5E). However, the Zn levels of live *Cg* continually decreased over time, presumably due to inhibition of phagolysosomal maturation and/or vacuolar Zn sequestration by *Cg*. In support of our hypothesis, the *Cg zrc1*Δ mutant strain showed an increased Zn^high^ PI^+^ dead population (Figure S5B), which might be caused by the defective ability to cope with toxic Zn concentrations (Figure S5C). Strikingly, upon treatment of BMDMs with IFNα or IFNβ, we observed that IFNs-I strongly inhibited the appearance of the Zn^high^ PI^+^ dead *Cg* population (Figures 5D and S5D), which is fully consistent with our confocal microscopy data. Accordingly, mCherry^+^*Cg* cells isolated from IFNβ-treated BMDMs showed reduced Zn acquisition when compared with fungal cells from untreated BMDMs (Figure 5F).

Further, we assumed that the IFNβ-mediated reduction in fungal Zn stress also translates into altered expression of Zn homeostasis genes in *Cg*. Therefore, we performed gene expression analysis from sorted *Cg*-infected BMDMs to exclude a possible interference from extracellular adherent *Cg* cells. Indeed, *Cg* isolated from IFNβ-treated BMDMs exhibited minor metallothionein expression of *MT-I,* showing that *Cg* encounters reduced intracellular Zn stress when phagocytosed by IFNβ-treated BMDMs (Figure 5G). Next, we infected BMDMs with live and heat-killed *Cg* upon pre-treatment with Bafilomycin A_1_ or DPI. Bafilomycin A_1_ is a selective inhibitor of vacuolar H^+^ ATPase, which prevents phagolysosomal maturation (Yamamoto et al., 1998), and DPI represents an NADPH oxidase inhibitor (Hancock and Jones, 1987). Strikingly, Bafilomycin A_1_ and DPI robustly reduced the generation of Zn^high^ PI^+^ *Cg* population (Figure 5H), showing that both phagolysosomal maturation and ROS production are required for potent Zn intoxication of *Cg*, presumably due to ROS-mediated Zn dissociation from Zn-MT complexes (Krężel and Maret, 2017). Taken together, these results show that BMDMs rapidly mobilize Zn to phagocytosed fungal cells to elicit Zn intoxication, which depends on phagolysosomal maturation and ROS generation. However, IFN-I signaling inhibits this antifungal immune defense mechanism, thus diminishing Zn stress *Cg* encounters in macrophages.

### MT1 and MT2 Are Required for Zn Intoxication of Pathogens

**Figure 6:**
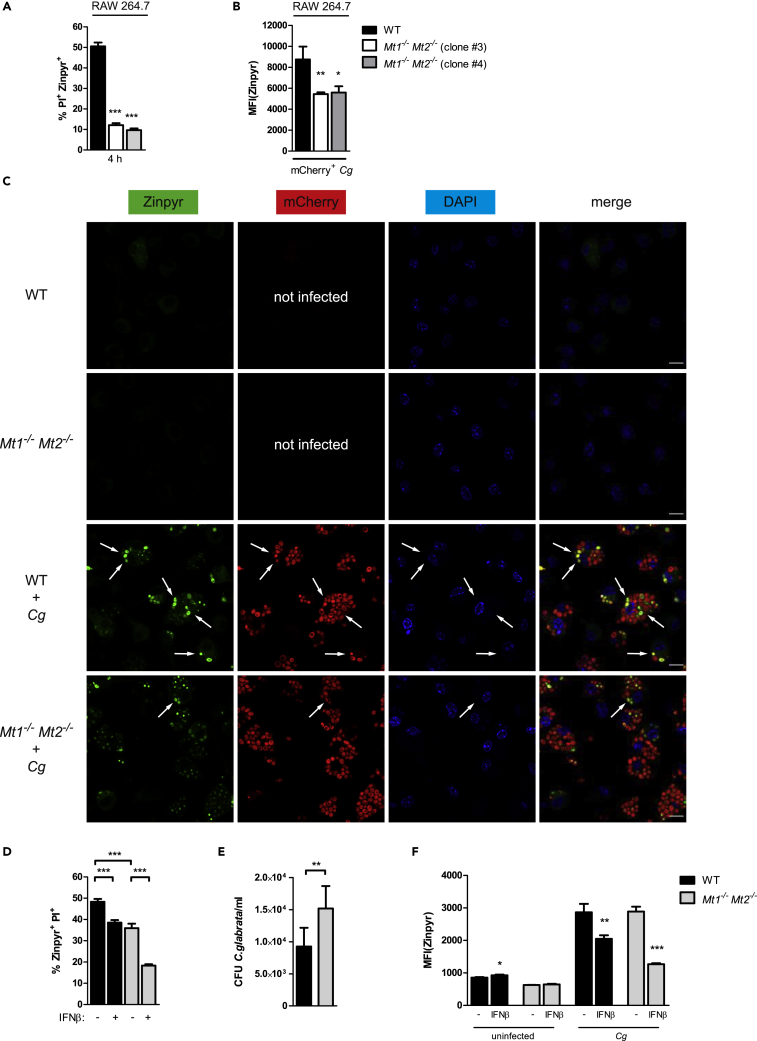
MT1 and MT2 Promote Fungal Zn Intoxication (A) Fungal Zn intoxication assay of *Cg* isolated from RAW264.7 cells after 4 h of infection. (B) Zinpyr-based fungal Zn acquisition of *Cg* isolated from RAW 264.7 cells after 6 h of infection. (C) Confocal microscopy analysis of Zn (Zinpyr; green), mCherry-expressing *Cg* (mCherry; red), and nucleus (DAPI; blue) in BMDMs after 4 h of *Cg* infection. (D) Fungal Zn intoxication assay of *Cg* isolated from untreated or IFNβ-treated BMDMs after 4 h of infection. (E) *In vitro* survival assay of *Cg* after 24 h interaction with BMDMs. (F) Zinpyr-assay of BMDMs untreated or IFNβ-treated during *Cg* infection for 8 h. Merge, overlay of all three channels. Arrows point at Zn^high^ yeast cells within infected BMDMs. The scale bar represents 10 μm. Data are representative of two (A–E) or three (F) independent experiments. Mean and SD are shown, ∗ p value < 0.05, ∗∗ p value < 0.01, ∗∗∗ p value < 0.001 (Student's t test). See also Figure S6.

Zinc-scavenging metallothioneins are key players for cytoplasmic Zn shuttling during intracellular Zn mobilization (Subramanian Vignesh and Deepe, 2017). Of note, our data revealed that IFNs-I robustly suppress metallothionein gene expression upon *Cg* infection both *in vitro* and *in vivo*. To investigate whether metallothioneins are involved in antifungal Zn intoxication, we undertook two different approaches. First, we used CRISPR/Cas9-generated *Mt1*^*−/−*^ *Mt2*^*−/−*^ double knock-out RAW 264.7 cells (Wu et al., 2017) for *Cg* infection. Strikingly, lack of MT1 and MT2 abrogated the generation of the Zn^high^ PI^+^ dead *Cg* population (Figure 6A), which was also accompanied with reduced Zn loading of *Cg* (Figure 6B). Second, we used primary bone marrow-derived macrophages from *Mt1*^*−/−*^ *Mt2*^*−/−*^ mice to verify the role of MT1 and MT2 (Rice et al., 2016). Interestingly, MT1 and MT2 were indeed required for Zn transport into *Cg*-containing vacuoles, because *Mt1*^*−/−*^ *Mt2*^*−/−*^ BMDMs revealed reduced amounts of Zn^high^*Cg*, whereby remaining fungal cells in *Mt1*^*−/−*^ *Mt2*^*−/−*^ BMDMs comprised the Zn^low^*Cg* population that displayed a remarkable vacuolar Zn staining (Figure 6C). These results were further supported by flow cytometry analysis, because lack of MT1 and MT2 in BMDMs reduced the generation of the Zn^high^ PI^+^ dead *Cg* population (Figure 6D), which was even more diminished upon IFNβ treatment. Moreover, the inhibition of Zn intoxication upon loss of MT1 and MT2 resulted in a decreased fungicidal activity of *Mt1*^*−/−*^ *Mt2*^*−/−*^ BMDMs (Figure 6E). However, as expected, the Zn burst upon *Cg* infection was unaffected in *Mt1*^*−/−*^ *Mt2*^*−/−*^ BMDMs (Figure 6F), because Zn mobilization within the cytoplasm is primarily controlled by ZIP and ZnT zinc transporters. Because MTs do not co-localize with mCherry^+^-*Cg*, we believe that MTs are not directly shuttled into *Cg*-containing phagosomes (Figure S6). Taken together, these results uncover an exciting role for MT1 and MT2 in antifungal immunity in addition to Zn sequestration (Subramanian Vignesh et al., 2013b). Once Zn is shuttled into the cytoplasm from intracellular compartments or the extracellular space, MT1 and MT2 act as Zn chaperones to facilitate subsequent Zn transport into *Cg*-containing phagosomes that drives fungal Zn intoxication and pathogen killing.

### JAK1 and IRF3 Are Engaged by IFNs-I for Zn Homeostasis Inhibition

**Figure 7:**
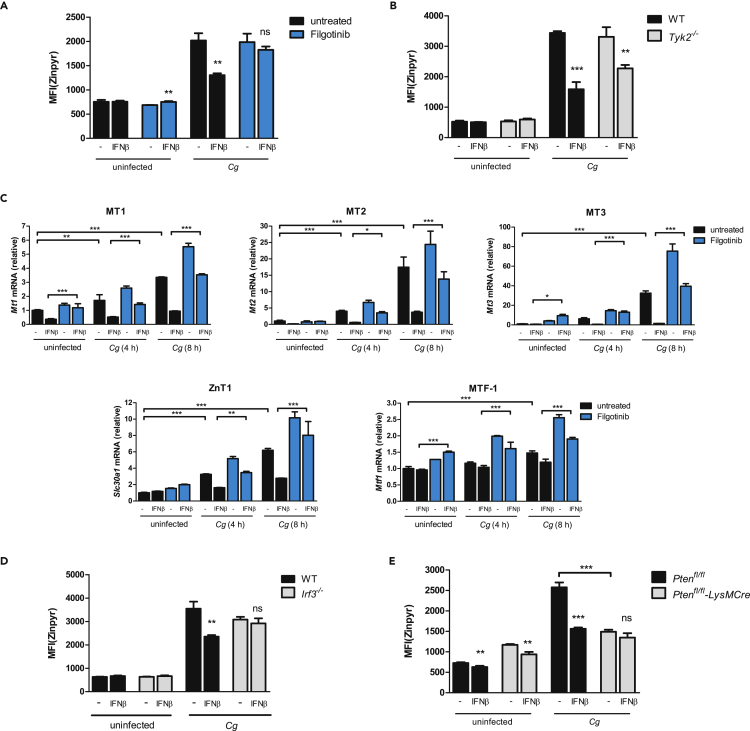
IFNs-I Engage JAK1 and IRF3 for Zn Homeostasis Dysregulation (A) Zinpyr-assay of untreated or IFNβ-treated BMDMs upon pre-treatment with Filgotinib and 8 h of *Cg* infection. (B) Zinpyr-assay of untreated or IFNβ-treated WT BMDMs upon *Cg* infection for 8 h. (C) RT-qPCR analysis of *Slc30a1*, *Mt3*, and *Mtf1* mRNA levels in untreated or IFNβ-treated WT BMDMs upon pre-treatment with Filgotinib and *Cg* infection (normalization to *Actb*). One-way ANOVA with Bonferroni's post hoc analysis. (D and E) Zinpyr assay of untreated or IFNβ-treated BMDMs upon *Cg* infection for 8 h. Data are representative of two (A–E) independent experiments. Mean and SD are shown; ∗ p value < 0.05, ∗∗ p value < 0.01, ∗∗∗ p value < 0.001; ns, not statistically significant; (A, B, D, and E) Student's t test; (C) one-way ANOVA with Bonferroni's post hoc analysis. See also Figure S7.

Next, we wanted to identify signal transduction components IFNs-I engage for subsequent inhibition of Zn homeostasis regulation. Upon binding of IFNs-I to IFNAR1, the receptor-associated kinases, Janus kinase 1 (JAK1) and TYK2, are phosphorylated and activated, triggering signaling pathways via STATs (via IRFs), PI3K, as well as MAPK signaling (Hervas-Stubbs et al., 2011). Strikingly, when we pre-treated BMDMs with the specific JAK1 inhibitor Filgotinib (GLPG0634) (Van Rompaey et al., 2013) prior to IFNβ stimulation, the inhibitory effect of IFNβ on Zn mobilization upon *Cg* infection was fully restored (Figure 7A). TYK2 was not required, because the inhibitory effect of IFNβ was observed upon loss of TYK2 (Figure 7B). Thus, JAK1 was exclusively engaged upon IFNβ stimulation. Further, JAK1 inhibition abolished the IFNβ-mediated transcriptional suppression of MT1, MT2, MT3, ZnT1, and increased transcription of MTF-1, the master regulator of key Zn homeostasis genes such as MTs (Figure 7C) (Günther et al., 2012). Notably, the inhibitory effects of IFNs-I bypassed STAT signaling (STAT1, STAT2, STAT3, STAT4, STAT5a/b, STAT6), p85α signaling, MAPK signaling (via MEK1), or the signal transducers IRF1 and IRF9 (Figures S7A–S7I). Strikingly, *Irf3*^*−/−*^ BMDMs were completely rescued from IFNβ-mediated Zn homeostasis inhibition, because their Zn burst was completely unaffected by IFNβ treatment (Figure 7D).

However, signaling via PTEN (Chen and Guo, 2017, Worby and Dixon, 2014) was necessary for an efficient Zn mobilization upon BMDM challenge with *Cg*, because *Pten*^*−/−*^ BMDMs failed to mount a robust Zn burst during *Cg* infection (Figure 7E). These results are in line with a previous report showing that PTEN also controls MTF-1 activation by associating with MTF-1 in the cytoplasm. Further, the PTEN protein phosphatase activity dephosphorylates MTF-1, which is required for regulation of target genes such as ZnT1 (Lin et al., 2012). Taken together, these data highlight that IFN-I stimulation engages JAK1 in a central role as “signaling relay” acting downstream of IFNAR1 to control signaling via IRF3 to regulate Zn homeostasis inhibition upon *Cg* infection. Moreover, BMDMs engage PTEN signaling for robust Zn mobilization, presumably via the regulation of MTF-1 activity.

### Dysregulation of Zn Homeostasis Suppresses the Generation of Fungicidal ROS

**Figure 8:**
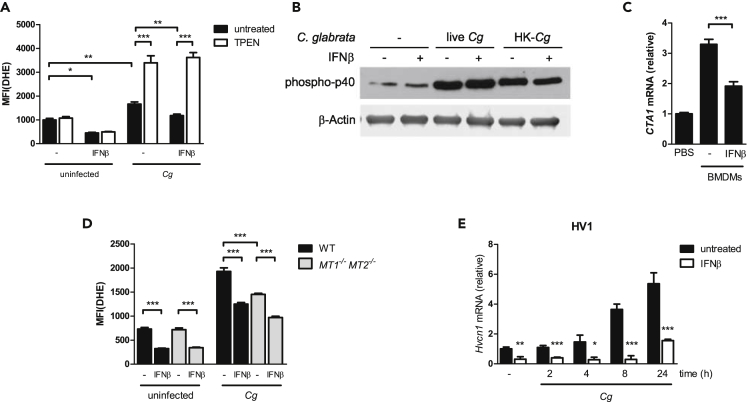
Fungicidal ROS Response in BMDMs Is Inhibited by IFNs-I (A) Detection of intracellular ROS by DHE in untreated or IFNβ-treated WT BMDMs challenged with *Cg* for 2 h and simultaneous TPEN (20 μM) treatment. (B) Immunoblot analysis of phospho-p40 activation in untreated or IFNβ-treated WT BMDMs challenged with live or heat-killed *Cg* for 60 min. (C) RT-qPCR analysis of *CTA1* mRNA levels in *Cg* upon infection of untreated or IFNβ-treated BMDMs for 2 h (normalization to *ACT1*). (D) Detection of intracellular ROS by DHE in untreated or IFNβ-treated BMDMs after 2 h of *Cg* infection. (E) RT-qPCR analysis of *Hvcn1* in WT BMDMs untreated or IFNβ-treated during *Cg* infection (normalization to *Actb*). Data are representative of two (A–E) independent experiments. Mean and SD are shown; ∗ p value < 0.05, ∗∗ p value < 0.01, ∗∗∗ p value < 0.001; (B, C, and E) Student's t test (A and D); one-way ANOVA with Bonferroni's post hoc analysis. See also Figure S8.

To ensure potent oxidative responses upon microbial challenge, macrophages must tightly control cytoplasmic Zn concentrations (Subramanian Vignesh et al., 2013b). Of note, cytoplasmic Zn ions suppress the NADPH oxidase-dependent ROS generation by inhibiting the hydrogen voltage-gated channel HV1 (*Hvcn1*) (DeCoursey et al., 2003). Consequently, upon infection with *Histoplasma capsulatum*, BMDMs upregulate MTs for subsequent Zn sequestration to sustain HV1 function and ROS generation (Subramanian Vignesh et al., 2013a). Because IFNs-I prevented *Mt* gene expression during *Cg* infection, we investigated whether IFN-I-mediated dysregulation of Zn homeostasis can also affect the antifungal ROS response. Indeed, BMDM stimulation with IFNβ suppressed the basal and *Cg*-induced amounts of intracellular ROS by about 30% (Figure 8A). However, phosphorylation of p40, a key regulatory subunit and activation marker of NADPH oxidase (Belambri et al., 2018), remained unaffected by IFNβ stimulation (Figure 8B). As a consequence of altered ROS generation, *Cg* sorted from IFNβ-treated BMDMs revealed a diminished oxidative stress response, due to the reduced expression of the ROS-detoxifying fungal catalase *CTA1* (Figure 8C) (Cuéllar-Cruz et al., 2008). Strikingly, TPEN treatment strongly increased ROS generation in untreated and IFNβ-treated BMDMs, showing that IFNβ-mediated ROS inhibition can be rescued by Zn chelation (Figure 8A). Consistently, the presence of exogenous Zn completely abrogated the *Cg*-induced ROS response (Figure S8A). In addition, Zn chelation by MTs was crucial for a robust ROS generation upon *Cg* infection, because both primary *Mt1*^*−/−*^ *Mt2*^*−/−*^ BMDMs (Figure 8D) and *Mt1*^*−/−*^ *Mt2*^*−/−*^ RAW 264.7 cells (Figure S8B) showed a diminished ROS response. Further, we detected substantially reduced *Hvcn1* mRNA levels in IFNα- as well as IFNβ-treated BMDMs, showing that IFNs-I, in addition to the previously observed reduction of *Mt* gene expression, also suppressed *Hvcn1* gene expression (Figures 8E and S8C). These data show that MT-mediated cytoplasmic Zn sequestration is crucial for the antifungal ROS response, which is suppressed by IFNs-I via a dual mechanism. First, IFN-I-mediated suppression of *Mt* gene expression results in BMDM failure to efficiently chelate Zn ions, and second, expression of ROS-promoting *Hvcn1* is dysregulated by IFNs-I.

### IFNs-I Modulate Splenic Macrophage Zn Pools upon Invasive *Cg* Infections

**Figure 9:**
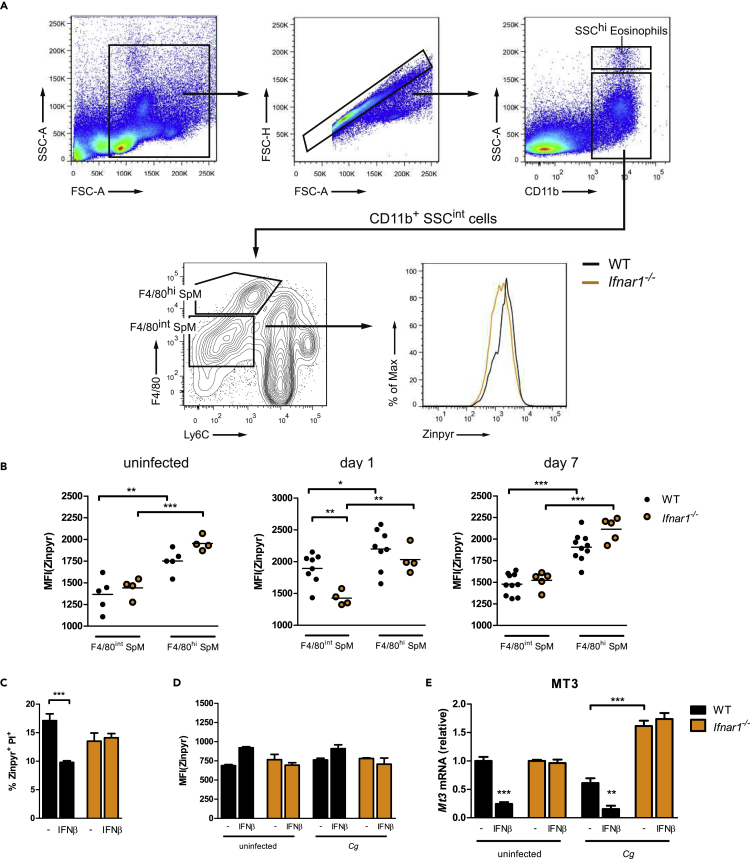
IFNs-I Suppress Zn Homeostasis Regulation in Splenic Macrophages during Invasive *C. glabrata* Infection *In Vivo* (A) Gating strategy for quantification of intracellular Zn levels in splenic macrophage subsets during *Cg* infection. Histogram of Zinpyr fluorescence by F4/80^int^SpMs from WT and *Ifnar1*^*−/−*^ mice. (B) Zinpyr-assay of splenic CD11b^+^ SSC^int^ F4/80^+^ macrophages isolated from WT and *Ifnar1*^*−/−*^ mice uninfected or IV infected with 5 × 10^7^ CFUs *Cg* for up to 7 days (n = 4–10 mice per group). Each symbol represents one mouse; horizontal bars indicate the calculated mean. (C) Fungal Zn intoxication assay of *Cg* isolated from untreated or IFNβ-treated *ex vivo* SpMs after 8 h of fungal challenge. (D) Zinpyr assay of untreated or IFNβ-treated *ex vivo* SpMs upon *Cg* infection for 8 h. (E) RT-qPCR analysis of *Mt3* mRNA levels in untreated or IFNβ-treated *ex vivo* SpMs upon and *Cg* infection for 8 h (normalization to *Actb*). Data are representative of two (B, C, and E) or three (D) independent experiments. Mean and SD are shown, ∗ p value < 0.05, ∗∗ p value < 0.01, ∗∗∗ p value < 0.001 (Student's t test).

Tissue-resident macrophages in the spleen represent a heterogeneous phagocyte population, which exert crucial defense mechanisms during systemic infections by microbial pathogens (Borges da Silva et al., 2015a, Borges da Silva et al., 2015b). Our data clearly show that IFN-I signaling modulates the splenic transcriptional regulation of Zn homeostasis genes and that Zn intoxication of pathogens by macrophages represents an antifungal defense strategy. Therefore, we wanted to monitor cellular Zn homeostasis alterations in splenic macrophage populations upon systemic *Cg* infection. After harvesting spleens from WT and *Ifnar1*^*−/−*^ mice, two populations of CD11b^+^ SSC^int^ splenic macrophages (SpMs) were identified: an F4/80^int^ SpM population and an F4/80^hi^ SpM population (Figure 9A). Of note, F4/80^int^ and F4/80^hi^ SpMs show intermediate to high CD11c expression (Riedelberger et al., 2020). Indeed, previous studies reported that red pulp macrophages and splenic DCs, as well as a skin DC subset, share a common phenotype of CD11c and F4/80 co-expression (Borges da Silva et al., 2015a, Borges da Silva et al., 2015b, McLachlan et al., 2009).

In uninfected mice, the lack of *Ifnar1*^*−/−*^ did not affect intracellular Zn levels in F4/80^int^ and F4/80^hi^SpMs, although F4/80^hi^SpMs revealed higher basal Zn acquisition (Figure 9B). Strikingly, at day 1 after *Cg* challenge, F4/80^int^SpMs from *Ifnar1*^*−/−*^ mice showed reduced Zn levels when compared with WT SpMs, an effect that was not observed in F4/80^hi^SpMs. These results are consistent with our *in vitro* data showing the double-edged effects of IFN-I signaling on Zn homeostasis. Thereby, BMDM treatment with IFNα/IFNβ suppressed the Zn burst upon *Cg* infection. However, basal IFNAR1 signaling was still required for cytoplasmic Zn mobilization, because *Ifnar1*^*−/−*^ BMDMs failed to trigger a Zn burst (Figures 2E and S2D). Therefore, we propose that F4/80^int^SpMs from *Ifnar1*^*−/−*^ mice fail to upregulate intracellular Zn levels upon systemic *Cg* infection due to the lack of basal IFNAR1 signaling. Of note, at day 7 of *Cg* challenge, F4/80^int^SpMs in WT and *Ifnar1*^*−/−*^ mice exhibited similar Zn levels (Figure 9B). Taken together, Zn mobilization in splenic macrophages upon systemic *Cg* infection represents a swift but transient response process, for which F4/80^int^ SpMs require basal IFNAR1 signaling.

To investigate the antifungal effector functions of SpMs in more detail, we cultivated primary splenic macrophages *ex vivo*. Strikingly, in line with our *in vitro* BMDM data, IFNβ-treated SpMs showed reduced fungal Zn intoxication after 8 h of *Cg* infection, which was fully rescued in *Ifnar1*^*−/−*^SpMs (Figure 9C). However, *Cg*-infected SpMs failed to mount a robust Zn burst (Figure 9D), suggesting that SpMs do not rely on extensive intracellular Zn mobilization as observed before in BMDMs. Again, and seen in the microarray data, IFNβ suppresses *Mt3*expression, whereby *Ifnar1*^*−/−*^SpMs show increased *Mt3* transcript levels (Figure 9E). Importantly, IFNβ promoted fungal survival and fitness in splenic macrophages, because IFNβ-treated SpMs revealed elevated intracellular replication of *Cg* (Riedelberger et al., 2020). In conclusion, as for other microbial infections, IFN-I signaling is detrimental for the host during *Cg* infections, because it also dysregulates the spatiotemporal Zn distribution in macrophages, thereby inadvertently suppressing other antifungal defense mechanisms that enable increased fungal fitness and immune evasion.

## Discussion

We have reported that IFN-I signaling is detrimental for the host during *Cg* infection by promoting fungal persistence in various organs (Bourgeois et al., 2011), thereby IFNs-I dysregulating host iron homeostasis, leading to unrestricted exploitation of intramacrophage iron pools by *Cg* (Riedelberger et al., 2020). Although confirming reports have been scarce, iron and Zn homeostasis in mammalian cells might be tightly intertwined and co-regulated, as reported before by our group for *Saccharomyces cerevisiae* (Landstetter et al., 2010). Further, reports showing an involvement of IFNs-I in Zn homeostasis regulation are limited, especially under infectious conditions (Guevara-Ortiz et al., 2005, Van Miert et al., 1990, Morris and Huang, 1987, Read et al., 2017, Sato et al., 1996). Here, we provide an in-depth mechanistic view of how IFN-I-mediated immune responses trigger a dysregulation of Zn homeostasis and spatiotemporal metal ion distribution in macrophages during *Cg* infection. IFN-I signaling diminishes several antifungal defense mechanisms at the cellular as well as organ level. First, we uncover that primary macrophages utilize MT1/MT2-mediated Zn intoxication to eradicate an intracellular fungal pathogen. However, host IFNs-I impair this antimicrobial defense by preventing Zn shuttling to *Cg*-containing phagolysosomes. Second, Zn homeostasis dysregulation by IFNs-I debilitates a potent fungicidal ROS response. Third, upon invasive *Cg* infection, IFNs-I suppress the transcriptional regulation of Zn homeostasis genes in the spleen and affect the cellular Zn regulation in splenic macrophages.

Zn intoxication has not been recognized as fungicidal mechanism to clear the intracellular pathogen *Cg*. However, our data demonstrate that MT1 and MT2 adopt a crucial role in antifungal immunity. Zn mobilization by Zn transporters, and MT1 and MT2, acting as Zn chaperones for Zn shuttling into *Cg*-containing phagolysosomes, drives Zn intoxication of phagosomally entrapped fungal cells. Interestingly, this process requires phagolysosomal maturation and ROS generation, presumably due to ROS-mediated dissociation of Zn from Zn-MT complexes (Krężel and Maret, 2017). The involvement of MTs in Zn intoxication during mycobacterial infections has been proposed but experimental evidence has been lacking (Botella et al., 2011). However, beautiful work shows that MT1 and MT2 are required for intracellular Zn sequestration by shuttling Zn away from phagolysosomes that contain *H. capsulatum* (Subramanian Vignesh et al., 2013a). In contrast, MT3 facilitates the access of *H. capsulatum* to labile Zn pools in macrophages, inadvertently leading to fungal Zn acquisition and persistence (Chowdhury et al., 2019, Subramanian Vignesh et al., 2016) Therefore, our data further highlight the function of MT1 and MT2 as central hubs that control the bidirectional Zn shuttling into or out of phagolysosomes during fungal infections. How MTs modulate the bivalent zinc transport into/out of pathogen-containing phagosomes for antimicrobial zinc intoxication or pathogen zinc starvation remains elusive. Potential mechanisms have been proposed, including direct MT shuttling into the phagosome for zinc release, MT binding to phagolysosomal membranes and ZIP/ZnT-mediated Zn transport, as well as simultaneous engulfment of extracellular zinc-bound MTs with the pathogen upon phagocytosis (Subramanian Vignesh and Deepe, 2017). It is also tantalizing to speculate that autophagosomal fusion with lysosomes could play a role in this phagosomal Zn delivery.

However, IFN-I responses suppress Zn intoxication of pathogens by transcriptional downregulation of Zn transporters and MTs, thereby promoting fungal fitness and survival. Further, we show that the receptor-associated JAK1, which is exclusively engaged by IFNs-I to transduce inhibitory signals via IRF3, enhances the dysregulation of Zn homeostasis. Interestingly, IFN-I signaling operates as double-edged sword for Zn intoxication, because IFNs-I suppress this antifungal defense mechanism, but basal, constitutive IFNAR1 signaling is required to mount a robust Zn burst. Resting immune cells rely upon basal IFNAR1 signaling by constitutively producing or responding to IFNs-I for the proper regulation of numerous cell functions (Gough et al., 2012). However, such functions can be suppressed by massive IFN-I production and overshooting IFN-I responses, which could even induce opposing signaling effects downstream of IFNAR1 receptor signaling (Gough et al., 2012, Hertzog and Williams, 2013, Schreiber and Piehler, 2015). In addition, although not affected by IFNs-I, we show that PTEN signaling is critical for Zn intoxication. PTEN is crucial for a potent Zn burst during *Cg* infection, presumably due to its involvement in MTF-1 activation as previously described (Lin et al., 2012). Thus, these data provide an in-depth mechanistic view how immune cells exploit Zn intoxication for clearing pathogens. Moreover, the dysregulation of Zn homeostasis by IFN-I signaling appears as an additional role of pro-inflammatory IFNs-I and likely plays a role in most, if not all, known functions of IFN-I responses.

IFNs-I are involved in multiple infectious diseases by exerting a plethora of pleiotropic functions in immune surveillance. Whether these effects drive pathogen clearance or inadvertently promote pathogen persistence is highly context and model dependent (Kernbauer et al., 2013, McNab et al., 2015, Stifter and Feng, 2015). In viral infections, IFNs-I activate cytotoxic effector cells (NK cells, CD8+ T cells) to orchestrate lysis of virus-infected cells, boost the production of virus-neutralizing antibodies, and induce antibody class switching (Teijaro, 2016). However, in certain settings, IFNs-I can also increase viral persistence and immunopathology (Snell et al., 2017) by inducing chronic immunosuppression of T cells and by promoting apoptosis of epithelial cells (Davidson et al., 2014, Teijaro et al., 2013, Wilson et al., 2013). During bacterial infections, IFNs-I augment leukocyte recruitment by controlling cytokine/chemokine production and by inducing expression of antibacterial effector molecules such as IDO, iNOS, or guanylate-binding proteins (Boxx and Cheng, 2016, Huang et al., 2019, MacMicking, 2012). However, IFN-I responses are also immunosuppressive during infections with several intracellular pathogens, including *Listeria monocytogenes* and *M. tuberculosis.* Thereby, IFNs-I sensitize several immune cell types to bacterial virulence factors and elicit apoptosis, suppress macrophage activation by inducing the downregulation of IFNγR expression, inhibit the production of pro-inflammatory cytokines, or downregulate iNOS expression (Calame et al., 2016, Dhariwala and Anderson, 2014, Donovan et al., 2017, Snyder et al., 2017, Toledo Pinto et al., 2018). In *C. albicans* infections, IFNs-I promote antifungal immunity by controlling chemokine production, leukocyte migration, NK cell activation, and epithelial barrier functions (Biondo et al., 2011, delFresno et al., 2013, Domínguez-Andrés et al., 2017, Li et al., 2017, Li et al., 2019). In contrast, exaggerated IFN-I responses reveal detrimental effects such as inhibition of inflammasome activation and inadvertently drive hyperinflammation and immunopathology during Candida spp infections (Bourgeois et al., 2011, Gabrielli et al., 2015, Guarda et al., 2011, Jensen et al., 1992, Majer et al., 2012, Stawowczyk et al., 2018, Riedelberger et al., 2020). Further, patients suffering from chronic mucocutaneous candidiasis exhibit defective IFN-I responses and suppressed antifungal IL-17 immunity (Casanova et al., 2012, Liu et al., 2011, Smeekens et al., 2013, van de Veerdonk et al., 2011). Finally, IFNs-I reveal double-edged functions in fungal infections with *Aspergillus fumigatus* (Espinosa et al., 2017, Gafa et al., 2010, Loures et al., 2015, Ramirez-Ortiz et al., 2011, Romani et al., 2006, Seyedmousavi et al., 2018), *Cryptococcus neoformans* (Biondo et al., 2008, Sato et al., 2015, Sionov et al., 2015), and *H. capsulatum* (Inglis et al., 2010, Van Prooyen et al., 2016). Owing to our presented data, we propose that dysregulation of metal ion homeostasis by IFN-I signaling represents an unrecognized concept of IFN-I biology in infectious diseases.

In addition to altering Zn homeostasis, IFNs-I strongly control the potency of the ROS response by macrophages, which is a key innate antifungal defense utilized by most phagocytes (Belambri et al., 2018, Cachat et al., 2015, Hogan and Wheeler, 2014). IFNs-I modulate the expression of ZnT/ZIP transporters and MTs in uninfected BMDMs, leading to increased intracellular Zn levels. However, cytoplasmic, unbound Zn ions are well known to inhibit the function of the proton channel HV1, which is required to mount a robust ROS response (DeCoursey, 2016, DeCoursey et al., 2003, Subramanian Vignesh et al., 2013a). During ROS production upon pathogen recognition, phagocytes simultaneously induce the expression of antioxidative MTs to minimize deleterious side effects on host tissues (Ruttkay-Nedecky et al., 2013). In addition, MTs are simultaneously required to bind cytoplasmic Zn, thereby augmenting antimicrobial ROS responses (Subramanian Vignesh and Deepe, 2017). Thus, our work reveals a dichotomous function of Zn sequestration by MT1 and MT2 in antifungal immunity. First, phagolysosomal Zn transport for Zn intoxication and second, promotion of HV1 function for maximal ROS production.

The dual functions of Zn ions may seem paradoxical at first hand. However, Zn is an essential metal required by numerous fundamental cellular process (Ferreira and Gah, 2017), but owing to the potent toxicity, Zn levels and subcellular Zn pools must be subject to tight and dynamic control (Lonergan and Skaar, 2019). Indeed, intraphagolysosomal/cytoplasmic Zn ions have to be kept low due to the mentioned inhibitory effects on the ROS response. However, Zn ions within the phagolysosome are required for fungal Zn intoxication. Thus, we propose a model in which cellular Zn homeostasis represents a delicate balance, for which macrophages are obliged to dynamically regulate the spatiotemporal distribution of Zn to ensure maximal efficiency for fungal clearance. First, the generation of ROS represents a swift but only temporary antifungal mechanism at the phagocytosis step (Blanco-Menéndez et al., 2015, Frohner et al., 2009, Salvatori et al., 2018, Warnatsch et al., 2017, Wellington et al., 2009). MTs are then engaged for Zn sequestration to boost HV1 function. Although the vast majority of fungal cells are killed in this initial phase by the oxidative burst, a considerable amount of *Cg* cells employ immune evasion mechanisms to establish intracellular niches for fungal replication and survival (Kumar et al., 2019). Thus, as a second line of defense, macrophages utilize Zn intoxication to tackle *Cg* survivors. Once Zn transporters are expressed and localize to the phagolysosome, MTs operate as Zn shuttling system to drive Zn intoxication, reaching maximal efficiency approximately 4 h after macrophage infection. We propose that ZnT1 is crucial for phagolysosomal Zn accumulation, and our data fully support earlier results concerning the role of ZnT1 (Botella et al., 2011).

We also show that IFNs-I suppress Zn homeostasis genes *in vivo* during the splenic response to systemic *Cg* infections. Remarkably, *ZnT10*, *ZIP2*, *ZIP8*, as well as *Mt1* and *Mt2* expressions are strongly diminished by IFNAR1 signaling. Interestingly, our microarray results are consistent with *in vitro* primary macrophage experiments, where the downregulation of MTs by IFNs-I is seen. Thus, our data additionally demonstrate a pivotal role for MTs in the spleen during fungal infections. Of note, and as proposed, the observed IFN-I-mediated transcriptional alterations also translate into a temporary dysregulation of cellular Zn levels in splenic macrophages upon invasive *Cg* infection *in vivo*.

However, the IFN-I-mediated suppression of MTs is contrasting previous studies showing that IFNα induces hepatic MT expression in several animal models (Guevara-Ortiz et al., 2005, Morris and Huang, 1987, Sato et al., 1996) and humans (Friedman and Stark, 1985, Nagamine et al., 2005). Of note, the reported transcriptional induction of MTs was only transient and timely restricted. Because we have primed our macrophages overnight with IFNs-I, a possible explanation could be that long-term stimulation with IFNs-I facilitates MT suppression. Further, cell-type-specific effects and differences in cellular environments in varying anatomical locations might also contribute to the reported observations. Moreover, we have not looked into the liver response, which displays an entirely different immunological competence. Although tissue-resident macrophage populations such as Kupffer cells are highly abundant in liver tissues (Macpherson et al., 2016), their role in antifungal defense at large has not been explored (Xu and Shinohara, 2017). Of note, Kupffer cells do phagocytose circulating *C. albicans* and *C. neoformans* cells to prevent fungal dissemination and launch pro-inflammatory cytokine responses (Øverland et al., 2005, Sun et al., 2019).

Virulence of fungal pathogens requires a tightly balanced Zn homeostasis (Wilson and Deepe, 2019). For example, Zn transporters are transcriptionally regulated in the yeast *S. cerevisiae* by the Zap1 regulator (Ballou and Wilson, 2016). In *C. albicans*, Zrt1 and Zrt2 are required for Zn uptake in a pH-dependent manner, thereby contributing to fungal virulence (Crawford et al., 2018, Kim et al., 2008). This regulatory mechanism closely resembles fungal Zn uptake in *A. fumigatus* (Amich et al., 2009), suggesting that the pH-dependency of Zn transport has been evolutionary conserved in fungal pathogens (Wilson, 2015). When encountering Zn limitation during phagocytosis, *C. albicans* secretes the protein Pra1 to sequester host-cell-derived Zn ions. Subsequently, Pra1 re-associates with fungal cells via cell-surface-localized Zrt1 to mediate Zn delivery, thereby also contributing to endothelial tissue invasion (Citiulo et al., 2012). Pra1 also contributes to fungal cell enlargement during Zn limitation (Malavia et al., 2017). Interestingly, metal acquisition via uptake of secreted cation-binding proteins/metabolites is predominantly conserved for bacterial pathogens (Kramer et al., 2019, Neumann et al., 2017, Palmer and Skaar, 2016), highlighting a distinguished role for the Pra1-Zrt1 system in *C. albicans*. Although a conserved locus structure *aspf2-zrfC* appears to exist in *A. fumigatus* (Amich et al., 2010), the Pra1-Zrt1 interaction represents a confirmed but unique zincophore system in fungi (Gerwien et al., 2018, Lehtovirta-Morley et al., 2017). In contrast, *C. albicans* counteracts environmental Zn excess and toxicity by Zrc1-mediated Zn compartmentalization in intracellular zincosomes, which is also implicated in fungal virulence (Crawford et al., 2018).

Likewise, bacterial pathogens have evolved sophisticated defense mechanisms to specifically prevent Zn intoxication. For instance, *M. tuberculosis* and *E. coli* employ P_1_-type ATPases to facilitate cellular Zn efflux, whereas *S. typhimurium* relies on Salmonella pathogenicity island 1 (SPI-I) (Botella et al., 2011, Kapetanovic et al., 2016, Stocks et al., 2019). In contrast, our data show that *Cg* detoxifies Zn by sequestration into the vacuoles, although the fungal transporters remain elusive. Of note, the regulation of Zn homeostasis in *Cg* in general is little understood. Most data come from the non-pathogenic yeast *Saccharomyces cerevisiae* (Gerwien et al., 2018, Wilson and Deepe, 2019). The vacuole represents the major Zn storage site, because it is the default Zn sink in yeast. The Zn importers Zrc1 and Cot1 localize to vacuolar membranes and control Zn transport, engaging V-ATPase activity (MacDiarmid et al., 2000, MacDiarmid et al., 2002). Strikingly, vacuolar Zn concentrations can reach up to 100 mM, and Zn can be rapidly mobilized to provide Zn supply for multiple progeny cells from one Zn-loaded mother (Simm et al., 2007). Thus, we propose that Zn sequestration into vacuoles represents a nutritional immunity mechanism of phagolysosomally trapped *Cg* to buffer otherwise toxic Zn levels, thereby facilitating fungal fitness and immune evasion. Strikingly, we show that genetic ablation of the putative Zn transporter orthologue Zrc1 renders *Cg* cells unable to sequester Zn ions into the vacuole, thereby strongly reducing fungal survival in macrophages. This notion is strongly supported by a recent study, showing that failure to assemble the vacuolar ATPase in *Cg* triggers a vacuolar pH imbalance, increased susceptibility to Zn stress, and attenuated fungal fitness *in vivo* (Minematsu et al., 2019).

Owing to the pleiotropic functions of IFNs-I in pathogenic infections (McNab et al., 2015), additional mechanisms might account for the reduced fungal organ loads in *Ifnar1*^*−/−*^ mice (Bourgeois et al., 2011, Riedelberger et al., 2020). However, the IFN-I-mediated dysregulation of Zn homeostasis highlights a possible and as yet unrecognized therapeutic concept for disseminated fungal infections. Because the inhibitory effects of IFNs-I were entirely controlled by JAK1, Filgotinib (GLPG0634) or related compounds might represent a promising option in order to lower otherwise detrimental IFN-I responses. Further *in vivo* experiments will be required to test this notion for fungal pathogens. Strikingly, Filgotinib has been used for the successful treatment of IFN-I-driven chronic inflammatory conditions, including inflammatory bowel disease, ankylosing spondylitis, psoriatic arthritis, and rheumatoid arthritis (Genovese et al., 2018, van der Heijde et al., 2018, Mease et al., 2018, Pérez-Jeldres et al., 2019). Further, specific inhibitors targeting fungal Zn homeostasis could constitute an exciting therapeutic option (Li et al., 2018), especially for hard-to-treat invasive infections with *Cg* or *C. auris*, owing to their intrinsic antifungal drug resistance (Perlin et al., 2017, Pristov and Ghannoum, 2019). Indeed, EDTA, TPEN, and zinc-attenuating compounds that indirectly also target Zn-dependent fungal processes have been effectively used for fungal infections with *C. albicans* and *A. fumigatus* (Cohrt et al., 2018, Hachem et al., 2006, Hein et al., 2015, Laskaris et al., 2016).

### Limitations of the Study

Pro-inflammatory IFN-I signaling acts as a double-edge sword at multiple intersections of host-pathogen interactions (McNab et al., 2015). IFNs-I can provide both detrimental and beneficial immune surveillance in a pathogen-specific manner, because they engage in multiple signal transduction pathways. IFNs-I control a plethora of immune cell functions during infectious diseases and thus also affect nutritional immunity (Boxx and Cheng, 2016, Stifter and Feng, 2015, Teijaro, 2016). Therefore, IFN-I response mechanisms are of ultrahigh complexity and dynamics as they engage or activate often unrelated immune pathways. Thus, we suggest that IFNs-I likely exert additional effects during systemic *Cg* infections, which might contribute to fungal organ persistence, perhaps in an organ-dependent fashion. Moreover, *in vivo* data addressing Zn fluctuations in various organs or immune cells in an organ- and or pathogen-specific manner are technically extremely challenging, owing to the ultrafast kinetics underlying adaptive metal ion changes in either host or pathogens. However, our data provide compelling evidence that dysregulation of Zn homeostasis and suppression of Zn intoxication in macrophages via metallothioneins contributes to the deleterious effects of IFN-I immune surveillance during infections with a major intracellular fungal pathogen.

### Resource Availability

#### Lead Contact

Further information and requests for resources and reagents should be directed to and will be fulfilled by the Lead Contact, Karl Kuchler (karl.kuchler@meduniwien.ac.at).

#### Materials Availability

Plasmids and fungal strains generated in this study will be made available upon request.

#### Data and Code Availability

The microarray data are deposited at the National Center for Biotechnology Information Gene Expression Omnibus (GEO: GSE134016) and are freely available.

## Methods

All methods can be found in the accompanying Transparent Methods supplemental file.

## Supplemental Information

Document S1. Transparent Methods, Figures S1–S8, and Tables S2–S4

Table S1. Microarray Analysis of WT and *Ifnar1*^*−/−*^ Spleens during Systemic Cg Infection, Related to Figure 1 Microarray expression data are presented as log2 fold changes of the indicated conditions with Benjamini-Hochberg adjusted p-values (padj). Log2 intensity values are shown in the “Intensity” column.
