# Supplementary material for: Type I Interferons Ameliorate Zinc Intoxication of Candida glabrata by Macrophages and Promote Fungal Immune Evasion
Source: iScience. 2020 May 4;23(5):101121. doi: 10.1016/j.isci.2020.101121 (PMC7232100; doi:10.1016/j.isci.2020.101121)
Supplement: Document S1. Transparent Methods, Figures S1–S8, and Tables S2–S4 [file mmc1.pdf]

iScience, Volume *23*

Supplemental Information

Type I Interferons Ameliorate Zinc Intoxication of *Candida glabrata* by Macrophages and Promote Fungal Immune Evasion

Michael Riedelberger, Philipp Penninger, Michael Tscherner, Bernhard Hadriga, Carina Brunnhofer, Sabrina Jenull, Anton Stoiber, Christelle Bourgeois, Andriy Petryshyn, Walter Glaser, Andreas Limbeck, Michael A. Lynes, Gernot Schabbauer, Guenter Weiss, and Karl Kuchler

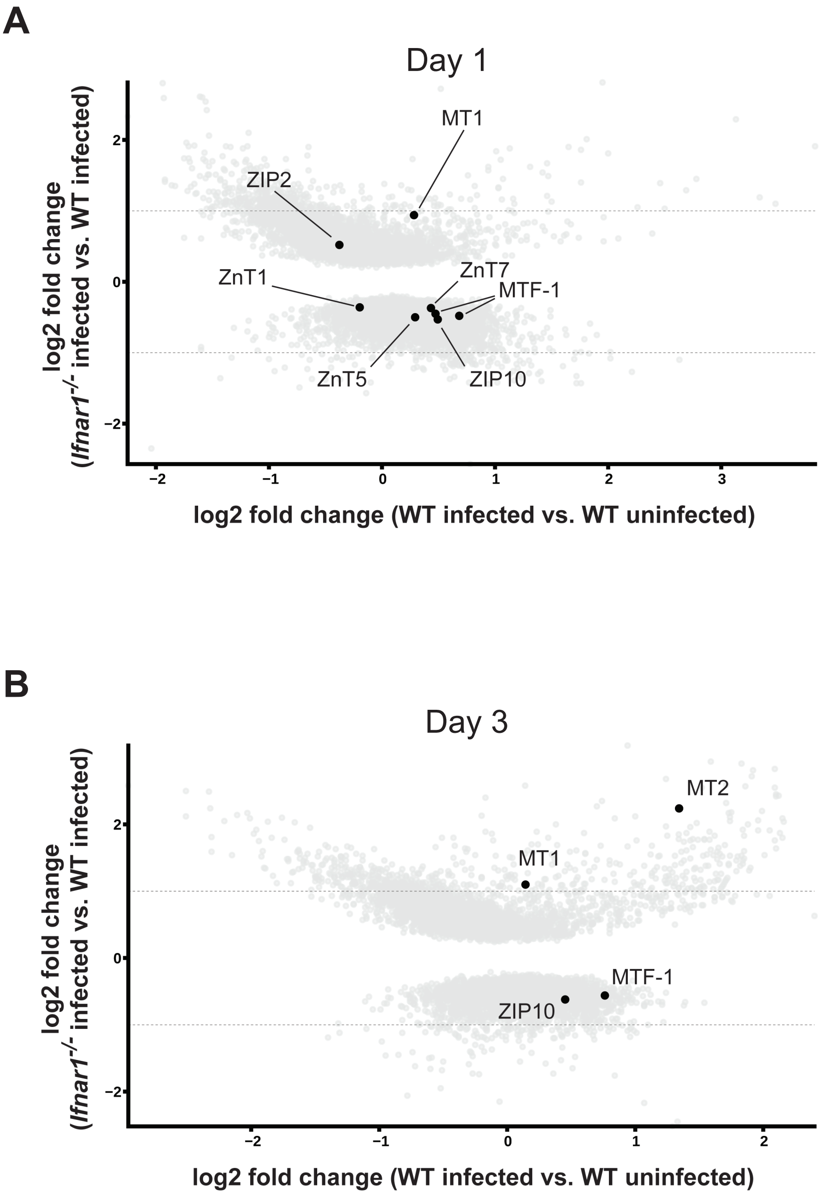

Figure S1. Microarray analysis of Zn homeostasis genes in *C. glabrata*-infected WT and *Ifnar1*$^{-/-}$ spleens. *Related to Figure 1.*

A-B) Scatter blots of DEGs from WT and *Ifnar1*$^{-/-}$ spleens at day 1 and day 3 of systemic *Cg* infection. Each dot represents one probe on the microarray and black dots (FDR < 0.05) correspond to Zn homeostasis-related genes.

DEG = Differentially Expressed Gene; FDR = False Discovery Rate

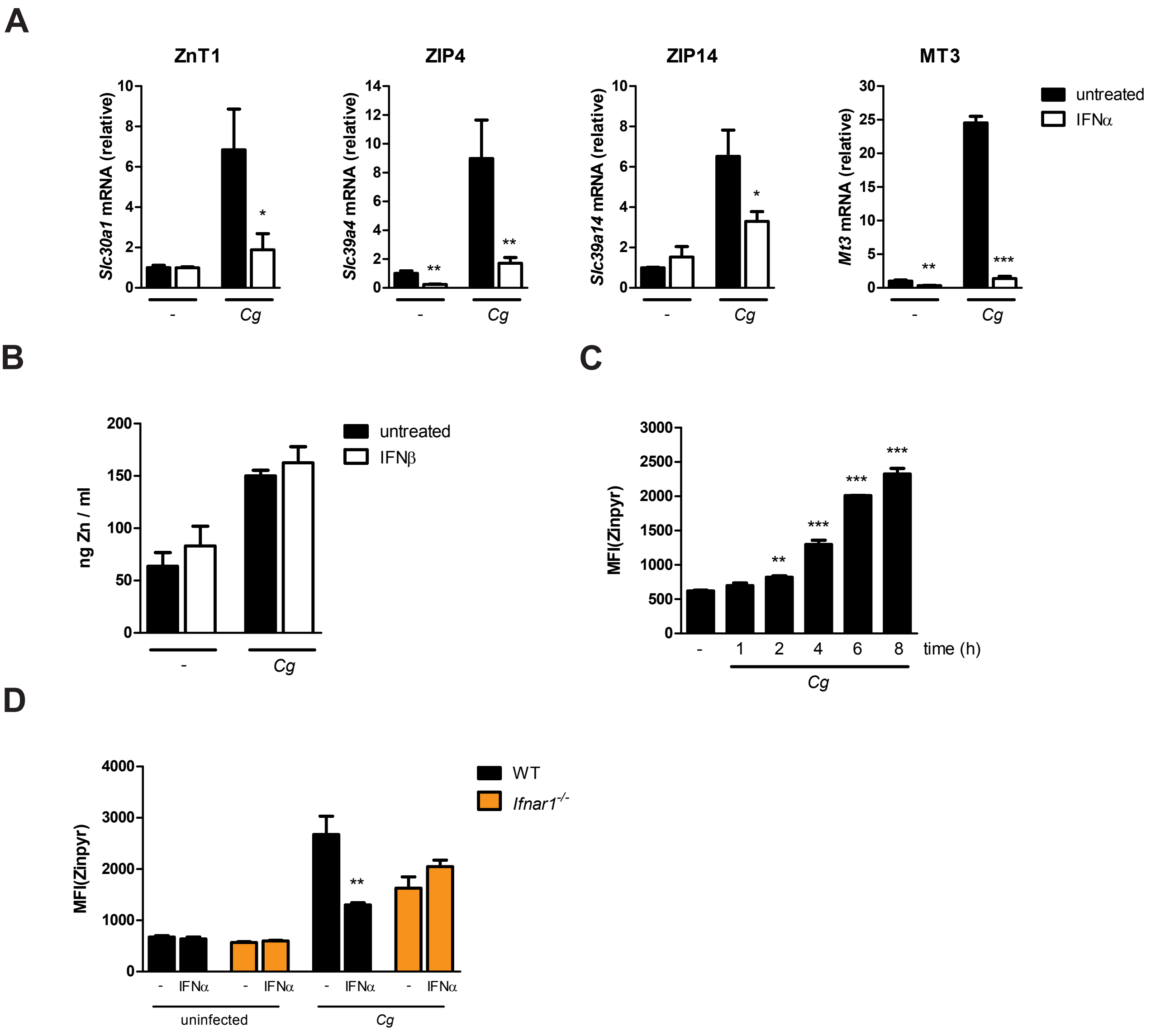

Figure S2. Regulation of Zn homeostasis by IFNα. *Related to Figure 2.*

A) RT-qPCR analysis of *ZnT1*, *ZIP4*, *ZIP14* and *Mt3* mRNA levels in untreated or IFNα-treated WT BMDMs challenged with *Cg* for 8 h (normalization to *Actb*).

B) Quantification of total Zn metal concentrations by ICP-MS in whole cell lysates from untreated or IFNβ-treated WT BMDMs infected with *Cg* for 8 h.

C) Zinpyr-assay of WT BMDMs infected with *Cg* for up to 8 h.

D) Zinpyr-assay of untreated or IFNα-treated BMDMs after 8 h *Cg* infection.

Data are representative of two (**A-D**) independent experiments. Mean and SD are shown, * p-value < 0.05, ** p-value < 0.01, *** p-value < 0.001 (**A-B,D**) Student's t-test (**C**) one-way ANOVA with Bonferroni's post hoc analysis.

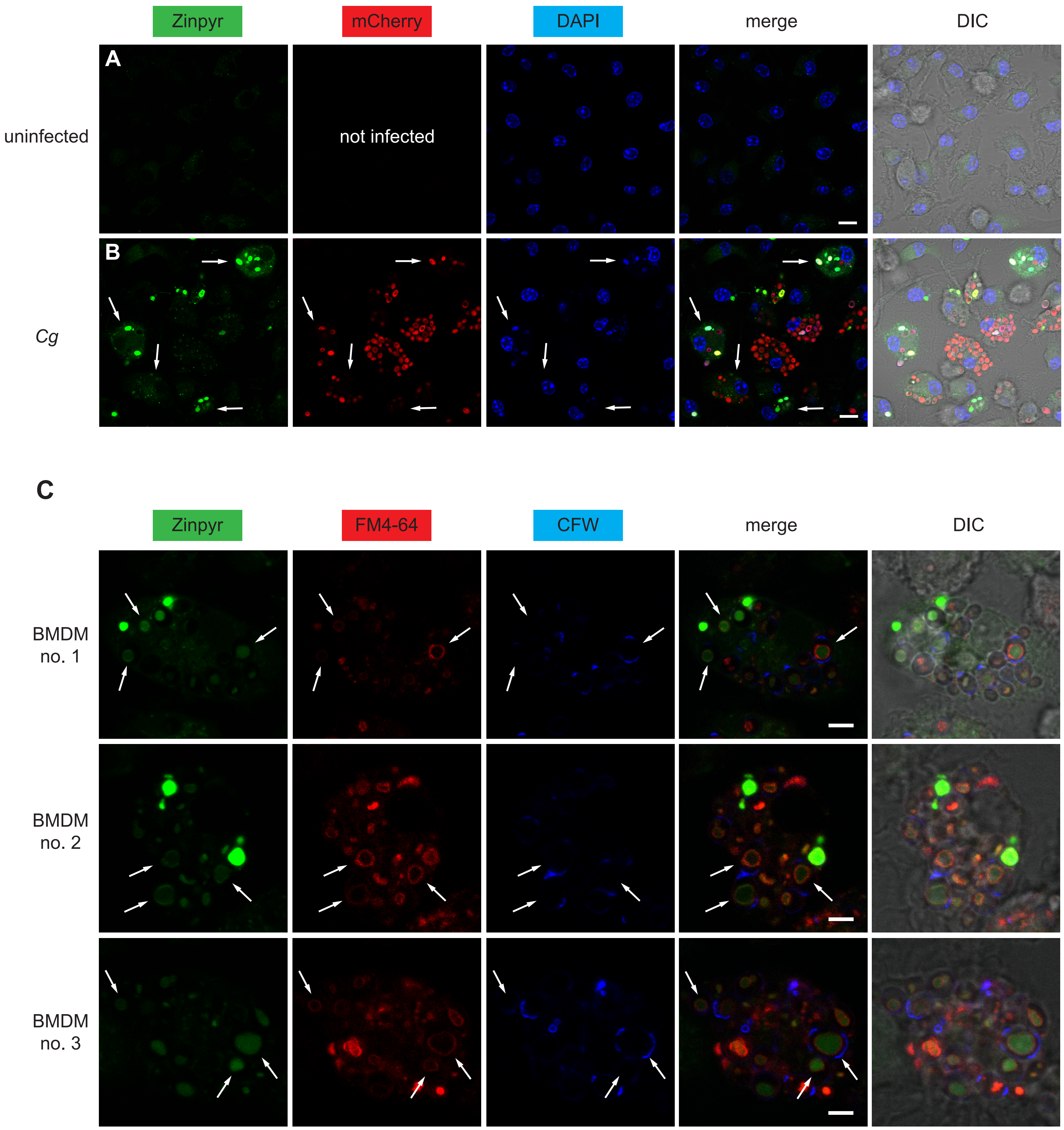

Figure S3. Zn localization in BMDMs during *Cg* infection. *Related to Figure 3 and 4.*
A-B) Confocal microscopy analysis of Zn (Zinpyr; green), mCherry-expressing *Cg* (red) and nucleus (DAPI; blue) in uninfected or *Cg*-infected WT BMDMs for 4 h.
C) Confocal microscopy analysis of three different WT BMDMs after 4 h *Cg* infection. Analysis of Zn (Zinpyr; green), vacuolar membranes of *Cg* (FM4-64; red) and Calcofluor-White-stained *Cg* (CFW; blue).
Merge, overlay of all three channels. DIC, Differential Interference Contrast. Arrows point at (**A-B**) BMDMs with increased cytoplasmic Zinpyr signal or at (**C**) vacuolar localization of Zn in *Cg*. The scale bar represents (**A-B**) 10 µm or (**C**) 5 µm. Data are representative of two (**A-C**) independent experiments.

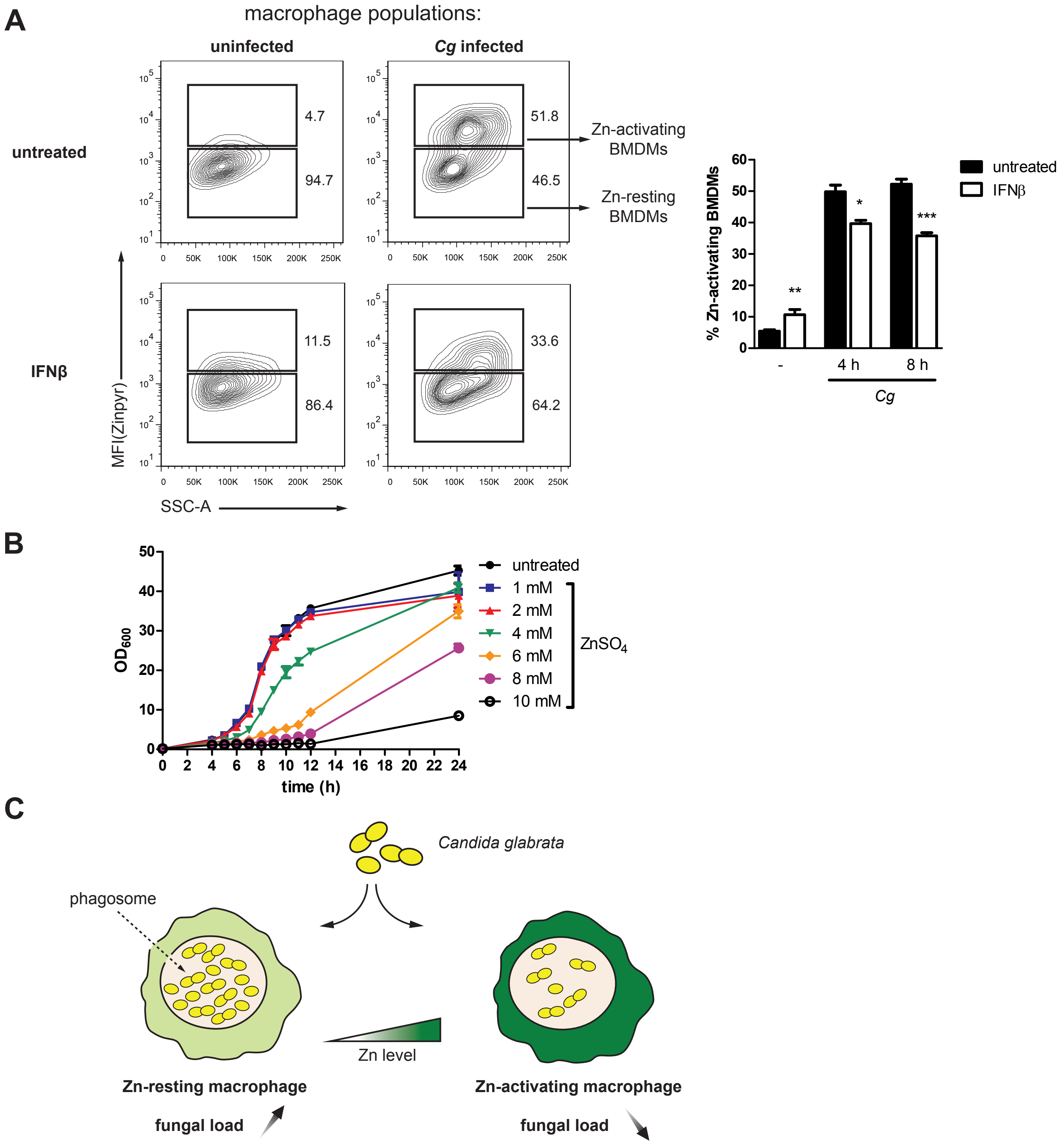

Figure S4. BMDMs and Zinc stress upon *Cg* infection. *Related to Figure 5.*

A) Flow cytometric quantification of Zn-activating WT BMDMs untreated or IFNβ-treated during *Cg* infection.

B) Quantification of fungal growth in liquid cultures upon incubation with varying Zn concentrations.

C) Graphical illustration. Macrophages separate into a Zn-resting and a Zn-activating population during *Cg* infection.

OD, Optical Density. Data are representative of two (**A-B**) independent experiments. Mean and SD are shown, * p-value < 0.05, ** p-value < 0.01, *** p-value < 0.001 (Student's t-test).

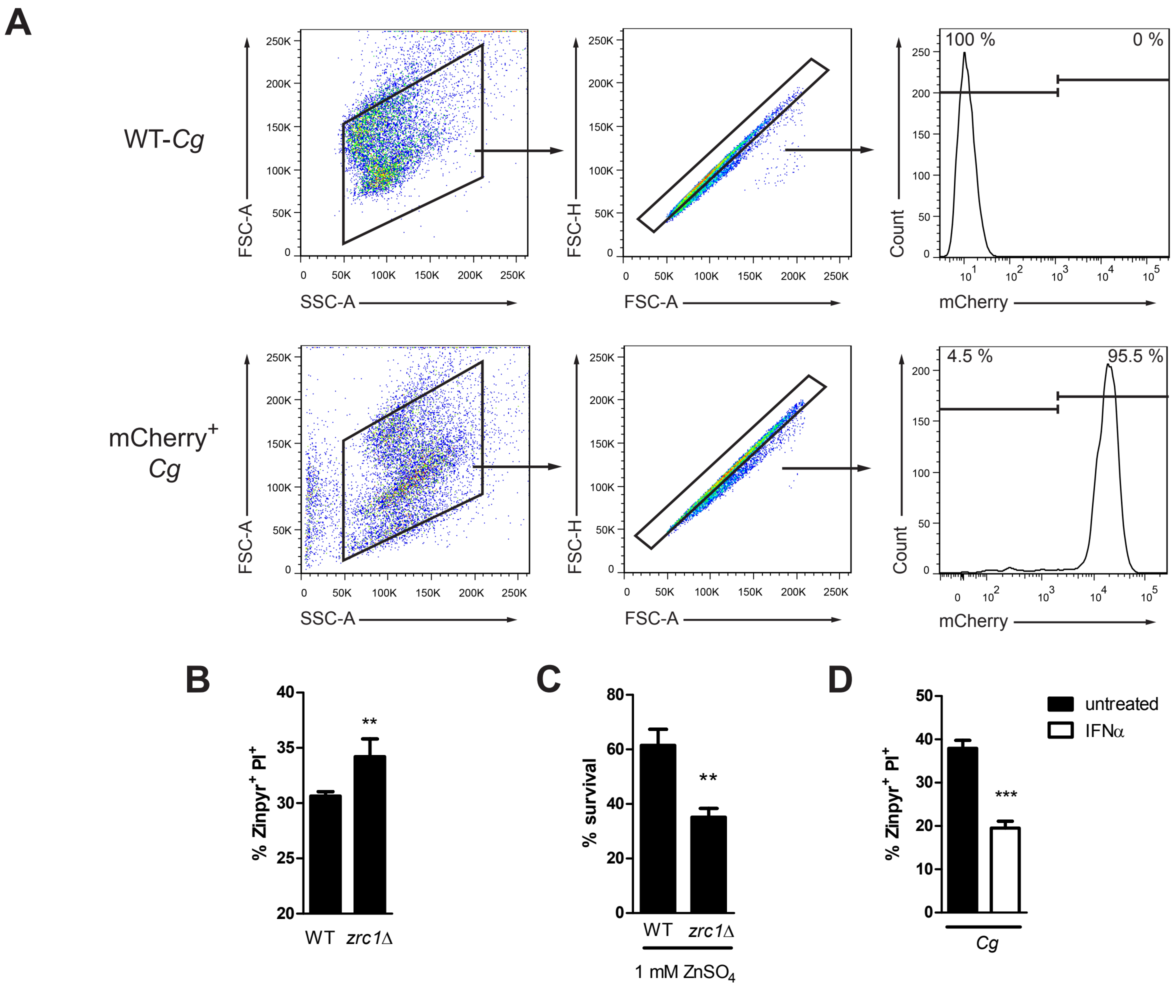

Figure S5. *Cg* and Zinc intoxication. *Related to Figure 5.*

A) Gating strategy for *Cg* identification. Via FSC/SSC discrimination, fungal cells can be accurately separated from smaller particles, since the entire *Cg* gate revealed mCherry^{+} cells.

B) Fungal Zn intoxication assay of WT and *zrc1*Δ *Cg* isolated from WT BMDMs after 8 h infection.

C) Fungal survival of WT and *zrc1*Δ *Cg* after 3 h incubation in 1 mM $ZnSO_4$/ddH_2O. Aliquots were serially diluted in PBS and plated on YPD plates for CFU enumeration.

D) Fungal Zn intoxication assay of *Cg* isolated from untreated or IFNα-treated WT BMDMs after 8 h infection.

Data are representative of two (**A-C**) or three (**D**) independent experiments. Mean and SD are shown, ** p-value < 0.01, *** p-value < 0.001 (Student's t-test).

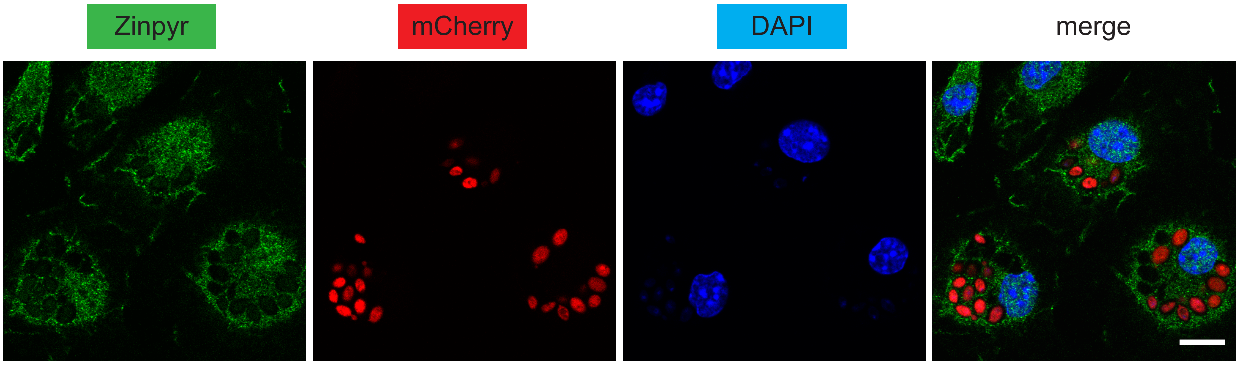

Figure S6. Nuclear and cytoplasmic localization of MTs in BMDMs during *Cg* infection. *Related to Figure 6.*
Confocal microscopy analysis of Zn (Zinpyr; green), mCherry-expressing *Cg* (red) and nucleus (DAPI; blue) in WT BMDMs after 4 h infection. Merge, overlay of all three channels. The scale bar represents 5 µm. Data are representative of two independent experiments.

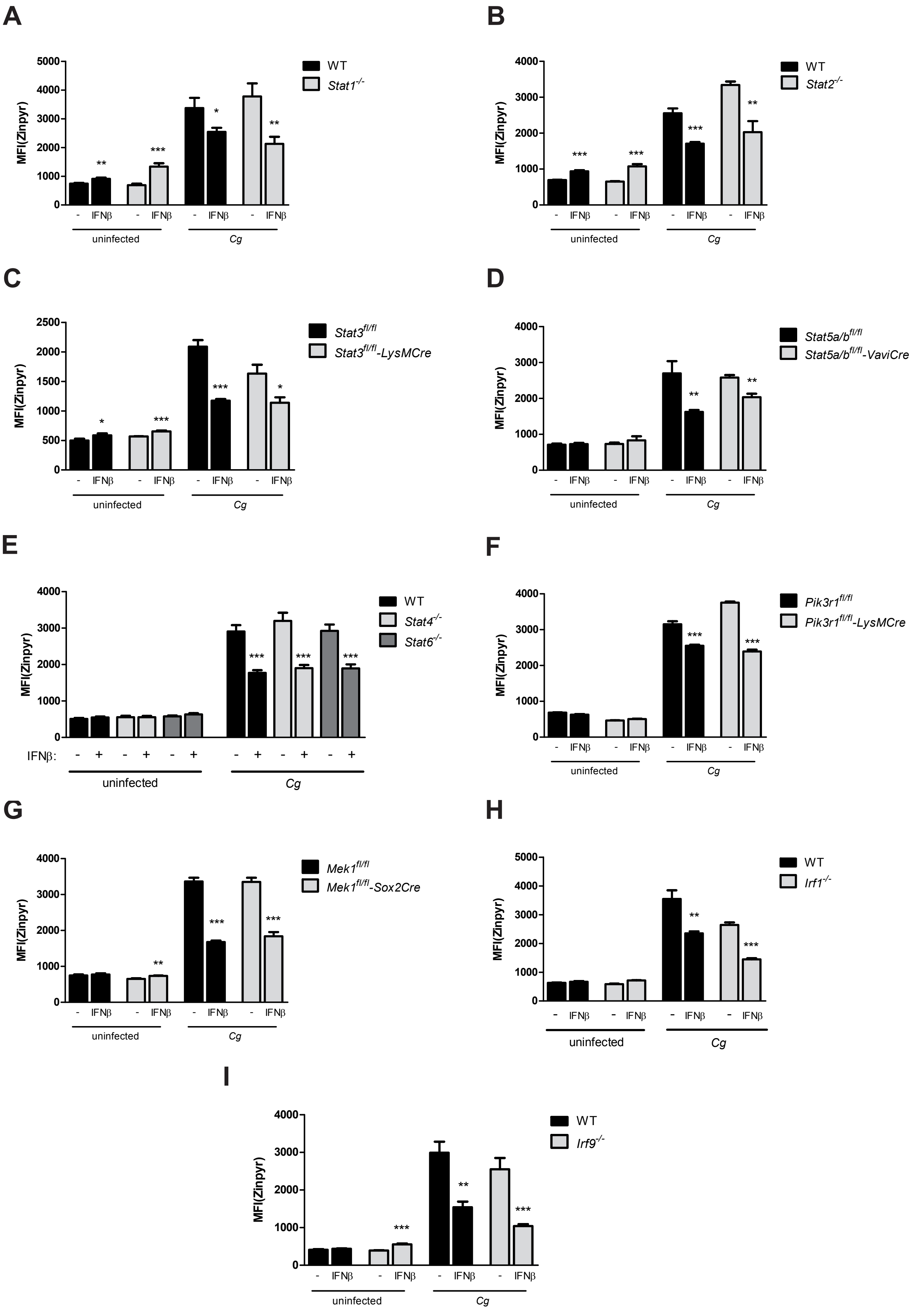

Figure S7. IFN-I-mediated dysregulation of Zn homeostasis is independent of various signaling pathways. *Related to Figure 7.*

A-I) Zinpyr-assay of untreated or IFNβ-treated BMDMs upon *Cg* infection for 8 h.

Data are representative of two (**A-I**) independent experiments. Mean and SD are shown, * p-value < 0.05, ** p-value < 0.01, *** p-value < 0.001 (Student's t-test).

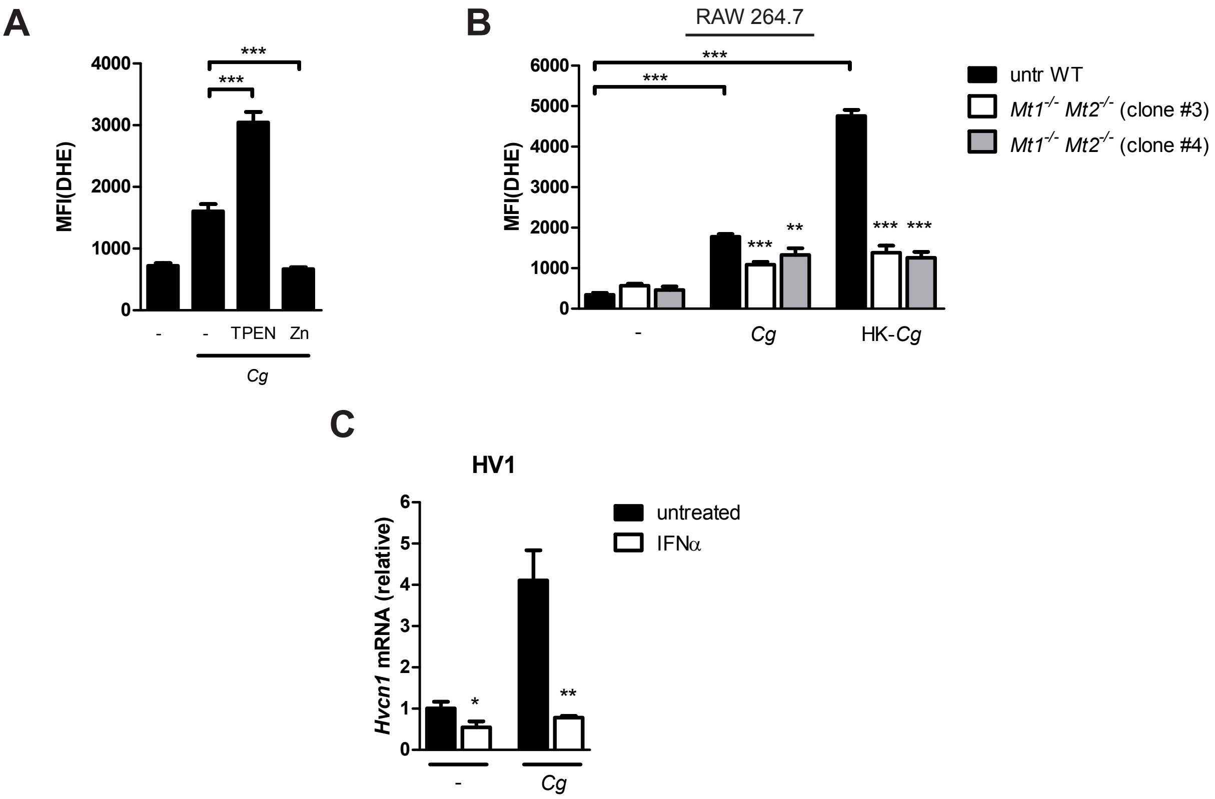

Figure S8. ROS response is modulated by Zn homeostasis. *Related to Figure 8.*

A) Detection of intracellular ROS by DHE. BMDMs were infected with *Cg* for 2 h and simultaneously treated with TPEN (20 μM) or $ZnSO_4$ (100 μM).

B) Detection of intracellular ROS by DHE in RAW 264.7 cells infected with live or heat-killed *Cg* for 2 h.

C) RT-qPCR analysis of *Hvcn1* in WT BMDMs untreated or IFNα-treated during *Cg* infection for 8 h (normalization to *Actb*).

Data are representative of two (**A-C**) independent experiments. Mean and SD are shown, * p-value < 0.05, ** p-value < 0.01, *** p-value < 0.001 (**A,C**) Student's t-test (**B**) one-way ANOVA with Bonferroni's post hoc analysis.

Table S2. List of Zn homeostasis-related genes involved in nutritional immunity. *Related to Figure 1.*

We recommend reviews focusing on nutritional immunity (Lopez and Skaar, 2018), Copper/Zinc intoxication (Sheldon and Skaar, 2019), Zinc homeostasis in infection and inflammation (Alker and Haase, 2018; Bonaventura et al., 2015; Gammoh and Rink, 2017; Gao et al., 2018; Sapkota and Knoell, 2018; Subramanian Vignesh and Deepe, 2016), gene regulation by MTF-1 (Günther et al., 2012) and metallothioneins in immunity (Rahman and Karim, 2018; Subramanian Vignesh and Deepe, 2017). Based on this literature, the microarray data were specifically analysed for Zn homeostasis pathways and the following Zn homeostasis-related genes have been overlayed with the microarray analysis results:

Gene	Synonym	UniGeneID
Mtf1	MTF-1	Mm.272397
Slc30a1	ZnT1	Mm.9024
Slc30a2	ZnT2	Mm.358876
Slc30a3	ZnT3	Mm.1396
Slc30a4	ZnT4	Mm.27801
Slc30a5	ZnT5	Mm.402215
Slc30a6	ZnT6	Mm.243943
Slc30a7	ZnT7	Mm.34550
Slc30a8	ZnT8	Mm.208831
Slc30a9	ZnT9	Mm.234455
Slc30a10	ZnT10	Mm.227117
Slc39a1	ZIP1	Mm.294709
Slc39a2	ZIP2	Mm.281343
Slc39a3	ZIP3	Mm.5353
Slc39a4	ZIP4	Mm.276829
Slc39a5	ZIP5	Mm.22983
Slc39a6	ZIP6	Mm.21688
Slc39a7	ZIP7	Mm.18556
Slc39a8	ZIP8	Mm.30239
Slc39a9	ZIP9	Mm.238279
Slc39a10	ZIP10	Mm.233889
Slc39a11	ZIP11	Mm.341021
Slc39a12	ZIP12	Mm.44662
Slc39a13	ZIP13	Mm.192375
Slc39a14	ZIP14	Mm.270647
Mt1	MT1	Mm.192991
Mt2	MT2	Mm.147226
Mt3	MT3	Mm.2064

Table S3. Oligonucleotides used in this study. *Related to Figure 2 and Figure 5-9.*

Primer name	Sequence (5’→3’)	
**Construction of *TDH3*-mCherry fusion**		
55_CgTDH3_SacI	actgctgagctcGTTTGATGACCACTGTCCAC	
53_CgTDH3_BamHI	actgctggatcctGTTCTTGGCAACGTGTTCAA	
35_CgTDH3_XhoI	actgctctcgagGGAAAAGTATCAACAGAGCATAG	
33_CgTDH3_KpnI	actgctggtaccCAATCTGCGTAGTAATGAGGAGTAA	
**Primers to verify genomic integration**		
5C_CgTDH3_mCherry	CAAGTACACTTCTGACTTGAAGATT	
mCherry_int_rev	TGAAGCGCATGAACTCCTTG	
***ZRC1* gene deletion**		
55IC_ZRC1	ttctttcctgcgttatcccctgattctgtggataaccgtaccatggACATAAGGTCGCATTTGTAGTATAC	Amplification of the *ZRC1* upstream region; lowercase letters represent the sequence overlap to the YEp352 backbone used for Gibson assembly; underlined sequence indicates a NcoI restriciton site
53IC_ZRC1	gaggggggggcccggtacccaattcgccctatagtgagtcgGTGCTAAATATTTGAGTCTGGTTC	Amplification of the *ZRC1* upstream region; lowercase letters represent the sequence overlap to the FRT-FLP-*NAT1*-FRT fragment used for Gibson assembly
35IC_ZRC1	tagtgagggttaattgcgcgcttggcgtaatcatggtcatCCGTTTTAAAATTTGAACGACC	Amplification of the *ZRC1* the downstream region; lowercase letters represent the sequence overlap to the FRT-FLP-NAT1-FRT fragment used for Gibson assembly
33IC_ZRC1	aacgcagaaaatgaaccggggatgcgacgtgcaagattaccatATGAATATCATCACTAAAACTGGC	Amplification of the *ZRC1* downstream region; lowercase letters represent the sequence overlap to the YEp352 backbone used for Gibson assembly
5C_ZRC1	GTGTGAGTGTGAGTGTGAGAG	Upstream integration check of the *ZRC1* deletion cassette
3C_ZRC1	GATGGTAGAAAATCTCATCGGTG	Downstream integration check of the *ZRC1* deletion cassette
LOG_ZRC1_fo	GATCACGGACATAGCCACGG	Validation of *ZRC1* gene deletion
LOG_ZRC1_rev	GCAGAAATTGTGGATGGAGTGG	Validation of *ZRC1* gene deletion
YEp_ic fwd	gtaatcttgcacgtcgcatcc	Amplification of the YEp352 backbone
YEp_ic rev	tacggttatccacagaatcaggg	Amplification of the YEp352 backbone
SATflipp_fwd	CGACTCACTATAGGGCGAATTGG	Amplification of the FRT-FLP-*NAT1*-FRT cassette from pSFS3b
SATflipp_rev	ATGACCATGATTACGCCAAGC	Amplification of the FRT-FLP-*NAT1*-FRT cassette from pSFS3b
hk3	CATCATCTGCCCAGATGCGAAG	Downstream integration check of the *ZRC1* deletion cassette
SATflipp_5C	TTTGGAACTAACGATGCATACGAC	Upstream integration check of the *ZRC1* deletion cassette
**Primers for RT-qPCR (mammalian cells)**		
ZIP1_Fw	ATGGAGTGAGACCCTCGGGA	
ZIP1_Rev	ACTGCATCTCCCCAATTCAG	
ZIP2_Fw	GAATGGGGAGGGACTCATGC	
ZIP2_Rev	CACAAGCCCCTTATGAGCCA	
ZIP3_Fw	AGAAGGATTGGCAGCAGCAT	
ZIP3_Rev	GTGCCACTCGACAGAACTCA	

ZIP4_Fw	CTTGGCTCTAGGCAAACCTG
ZIP4_Rev	AGTGTGGCCAGGTAATCGTC
ZIP5_Fw	CTGGCAGTCCTGTTCCTCAG
ZIP5_Rev	GACCCAGGTCCTCCTCTGAT
ZIP6_Fw	TGACCTTTGCCCTTTGGGTT
ZIP6_Rev	ATGGTGTCGCTGCATGGTAA
ZIP7_Fw	GAGAGGAGGAAACACTGGGC
ZIP7_Rev	GTTGTGTGCCAAGTCAGCAG
ZIP8_Fw	GAGACAATGCAAGGGGCTCT
ZIP8_Rev	CTTCGGGGCATTGAAGAGGT
ZIP9_Fw	TGTGTGACGCTCCTCACTTC
ZIP9_Rev	GCTGTTCCACTTCTCCACGA
ZIP10_Fw	GTTAAAAGCCGCCCCAACTC
ZIP10_Rev	CTGTAGCTCCGCGCTAGTTT
ZIP11_Fw	CAAGGTTACAGCTCCGTGGT
ZIP11_Rev	GTCTAAGATCCGCCTCTGCC
ZIP12_Fw	AACACAACCCAAGCCCAAGA
ZIP12_Rev	GGATGGTTGAGGGACCAGTG
ZIP13_Fw	CCCATGGTATGAGGCGGAAG
ZIP13_Rev	CAGTGGCAGAGGTGGCAG
ZIP14_Fw	CGTGGGAGCCAACTGATAAT
ZIP14_Rev	AACGGCCACATTTTCAACTC
ZnT1_Fw	GCTCTCGAGTTGGTCCTGTC
ZnT1_Rev	GCCTCATGGTGAGGTAGGAA
ZnT2_Fw	AGCCCGGTCCTTCTTAGGAT
ZnT2_Rev	GGATCCGGCTAGCTTCACTC
ZnT3_Fw	AGGCGTGGGAGATAGAGACT
ZnT3_Rev	TCCAAGGACCACTCGGACTC
ZnT4_Fw	TGAGTGGCACAACCTCTCAG
ZnT4_Rev	ACTGCATCTCCCCAATTCAG
ZnT5_Fw	TGTCCAAATGTGCCCGCTAT
ZnT5_Rev	CTCTGTGCTCTCCTGGTGTG
ZnT6_Fw	TTAATCGCGGACACACACCA
ZnT6_Rev	GCCCCATCCTGTTGATTCCA
ZnT7_Fw	GTGCCTGAACCTCTCTTTCG
ZnT7_Rev	TCAAAAACAGCCCATTGACA
ZnT8_Fw	TGTCCAAATGTGCCCGCTAT
ZnT8_Rev	TACTTCACACACACAACACCA
ZnT9_Fw	CTATCGTGCCTCACACCTGG
ZnT9_Rev	AGCCTCTTCAAGCCTTTCGT
ZnT10_Fw	GTCCTCCACATTTCCCCTGG
ZnT10_Rev	TTGGGGTTGGGGATTGTAGC
MT1_Fw	CACCAGATCTCGGAATGGAC
MT1_Rev	GTTCGTCACATCAGGCACAG
MT2_Fw	CCGATCTCTCGTCGATCTTC
MT2_Rev	ACTTGTCGGAAGCCTCTTTG
MT3_Fw	AGACCTGCCCCTGTCCTACT
MT3_Rev	CAGGGACACCCAGCACTATT
HV1_Fw	TCGAGCAGCTCTGGAAACTC
HV1_Rev	TGTCCTCGAAGGTCCGTTTG
MTF-1_Fw	TTCTCACAATTGGGCTGAGCA
MTF-1_Rev	ACCAGTCCGTTGTCATCCAC
b-Actin_Fw	CCTTCCTTCTTGGGTATGGA
b-Actin_Rev	ACGGATGTCAACGTCACACT
**Primers for RT-qPCR (fungal cells)**	
MT-I_Fw	AACGGTTGCTCCTGTCCAAA
MT-I_Rev	ACCGCACTTGCATTGTTCAC
CTA1_Fw	GGTCCAGCTCAACCATTCCA
CTA1_Rev	ACAAGTCTCTGGCTTGGACG

Act1_Fw	AATTGAGAGTCGCCCCAGAA
Act1_Rev	GGCTGGAACGTTGAAGGTTT

Table S4. Plasmids used in this study. *Related to Figure 4.*

Name	Description	Reference
pSFS3b	Donor plasmid for *NAT1*-Flipper used for Gibson assembly	(Tscherner et al., 2015)
YEp352-*SAT1*	Donor plasmid for *E. coli* replication origin and ampicillin resistance fragment for Gibson assembly	(Krauke and Sychrova, 2011)
YEp352-*NAT1*-Cg*ZRC1*urdr	*ZRC1* gene deletion plasmid generated via Gibson assembly	This study

Transparent Methods

Ethics statement

All animal experiments were evaluated by the Ethics Committee of the Medical University of Vienna and approved by the Federal Ministry for Science & Research, Austria (BMBWF-66.009/0436-V/3b/2019).

Mouse experiments

Ifnar1$^{-/-}$ (Müller et al., 1994), *Tyk2*$^{-/-}$ (Shimoda et al., 2000), *Stat1*$^{-/-}$ (Durbin et al., 1996), *Stat2*$^{-/-}$ (Park et al., 2000), *Stat3*$^{fl/fl}$ (Alonzi et al., 2002), *Stat4*^{em3Adiuj} (#028526; obtained from the Jackson Laboratory), *Stat5a/b*$^{fl/fl}$ (Cui et al., 2004), *Stat6*$^{-/-}$ (Kaplan et al., 1996), *Irf1*$^{-/-}$ (Reis et al., 1994), *Irf3*$^{-/-}$ (Sato et al., 2000), *Irf9*$^{-/-}$ (Kimura et al., 1996), *Mek1*$^{fl/f}$ (Catalanotti et al., 2009), *Pik3r1*$^{fl/fl}$ (Luo et al., 2005), *Pten*$^{fl/fl}$ (Suzuki et al., 2002), C57BL/6J-*Mt1*^{tm1Bri} *Mt2*^{tm1Bri} (Masters et al., 1994; Rice et al., 2016), *LysMCre* (Clausen et al., 1999), *VaviCre* (Boer et al., 2003) and *Sox2Cre* (Hayashi et al., 2002) mice have been described. Wild-type (C57BL/6J) and *Ifnar1*$^{-/-}$ mice were housed under specific pathogen-free conditions in the animal facility of the Medical University of Vienna/Max Perutz Labs Vienna. Mice breeding and maintenance was in accordance with ethical animal license protocols complying with the current Austrian law. Male and female WT and *Ifnar1*$^{-/-}$ mice (8-12 weeks old) were infected intravenously with 5 x 10^7 colony-forming units (CFUs) of *C. glabrata* (in 100 µl) per 25 g mouse weight. Throughout the infection, mice were monitored daily and killed by cervical dislocation at the respective time points.

Fungal Strains and Culture Conditions

Fungal strains used in this study included the wild-type *C. glabrata* strain ATCC2001, a *zrc1*Δ mutant strain and a mCherry-expressing *C. glabrata* strain. For fungal cultivation, rich YP medium (Yeast extract: BD Biosciences; Tryptone: AppliChem) supplemented with 2 % (w/v) glucose (Sigma-Aldrich) was used (Kaiser et al., 1994). *C. glabrata* was incubated at 70 °C at 800 rpm for 10 min (heat-killing) or for 1 min (heat-stressed cells). Heat-killed cells were checked via YPD plating for surviving fungal cells. To determine fungal growth by optical density (OD_{600}) measurement, logarithmic growing *C. glabrata* were adjusted to OD_{600} = 0.2 in YPD medium substituted with varying $ZnSO_4$ concentrations (Sigma-Aldrich) and grown at 30 °C and 220 rpm. For confocal microscopy, *C. glabrata* was stained with 25 µg/ml Calcofluor-white (Sigma-Aldrich) at room temperature for 10 min at 800 rpm in the dark. For vacuolar staining, *C. glabrata* was incubated in 1:250 FM4-64 (Thermo Fisher Scientific) in YPD medium at 30 °C for 1 h at 800 rpm in the dark.

Plasmid and *C. glabrata* Strain Construction

Oligonucleotides and plasmids used in this study can be found in Table S3 and S4. *C. glabrata* mCherry tagging was done via C-terminal fusion of *TDH3* (CAGL0G09383g) with mCherry based on a previously published strategy (Yáñez-Carrillo et al., 2015) using the pYC56 plasmid (Addgene) as a mCherry donor. Briefly, the last 485 bp of the *TDH3* coding sequence (CDS; excluding the stop codon) and 118 bp downstream (DR) of the open reading frame were PCR amplified and cloned into pYC56

using SacI, BamHI and KpnI, XhoI (all Thermo Fisher Scientific), respectively, yielding the final plasmid pYC56-TDH3urdr. To excise the final Tdh3-mCherry tagging cassette, pYC56-TDH3urdr was digested with SacI and KpnI and transformed into *C. glabrata* using electroporation as described earlier (Reuss et al., 2004). The integration of the mCherry tagging cassette into the *TDH3* locus was confirmed by colony PCR.

C. glabrata ZRC1 (CAGL0K07392g) gene deletion was done in the ATCC2001 strain background using a modified *SAT1* flipper technique (Reuss et al., 2004; Tscherner et al., 2015). Briefly, a YEp352-*NAT1*-Cg*ZRC1*urdr plasmid was generated via a Gibson assembly approach (Gibson et al., 2009). Therefore, approximately 500 bp fragments of up- and downstream regions of the *ZRC1* coding sequence (CDS) were PCR amplified and fused with a FRT-FLP-*NAT1*-FRT cassette generated from pSFS3b (Tscherner et al., 2012) and the backbone from YEp352-*SAT1* containing the *E. coli* replication origin and an ampicillin resistance marker. Gibson assembly was done using a 2x Gibson assembly master mix (New England Biolabs) and 10 ng/kb of agarose gel-purified fragments. The generated YEp352-*NAT1*-Cg*ZRC1*urdr plasmid was digested with FastDigest NcoI and PvuI (both ThermoFisher Scientific) and transformed into *C. glabrata* using electroporation as described earlier (Reuss et al., 2004). The up- and downstream integration, as well as the deletion of *ZRC1* were confirmed by colony PCR.

For Colony PCR, a single colony of *C. glabrata* was resuspended in 50 µl H_2O and incubated for 10 minutes at 95 °C. Cell debris were spun down and 5 µl of the supernatant were used for PCR using the DreamTaq Green DNA Polymerase (Thermo Fisher Scientific) in accordance to the manufacture's protocol as previously described (Tscherner et al., 2015).

Microarray Sample Preparation and Analysis

WT and *Ifnar1*$^{-/-}$ mice were intravenously infected with *C. glabrata* (see above). Spleens were collected at day 1, 3, 7 and 14 post-infection and stored in RNA*later*™ Stabilization Solution (Thermo Fisher Scientific). Organs were homogenized in 1.5 ml RNA lysis buffer (Promega) with occasional cooling on ice by using an Ika T10 basic Ultra-Turrax homogenizer (IKA). For RNA isolation, the SV Total RNA Isolation System (Promega) was used according to the manufacturer's instructions.

RNA quality was checked on RNA 6000 Nano chips (Agilent) using a Bioanalyzer 2100 (Agilent). The Low Input Quick Amp Labeling Kit (one-color) (Agilent) was used to generate fluorescent cRNA. The amplified cyanine 3-labeled cRNA samples were then purified using SV Total RNA Isolation System (Promega) and hybridized to the SurePrint G3 Mouse GE 8x60K microarray (Agilent). Microarray slides were washed and scanned with a DNA Microarray Scanner (Agilent), according to the standard protocol of the manufacturer. Information from probe features was extracted from microarray scan images using the Feature Extraction software v10.7.3 (Agilent).

Further analyses were performed using R Bioconductor (Gentleman et al., 2004). The raw intensities were imported into Bioconductor using spot weighting and further processed with the limma package (Smyth, 2004). Quality Controls were performed using the arrayQualityMetrics package (Kauffmann et al., 2009). To reduce the effects of outlier arrays, arrays were weighted using the arrayWeights function of limma. Normalization between arrays was performed using the quantile method and a linear model was fitted. P-values were adjusted for multiple testing using the Benjamini

& Hochberg method. Log2 intensities for *Ifnar1*$^{-/-}$ and WT samples were normalized to uninfected controls first and subsequently differentially expressed genes in *Ifnar1*$^{-/-}$ versus WT samples were determined for each day. After filtering out of low-intensity probes (average log2 intensity > 6), cutoffs for differential expression were set to a minimum 2-fold up- or down regulation and a maximum adjusted p-value of 0.05 with at least one probe matching these criteria. Normalized log2 intensity values were used to perform gene set enrichment analysis using gene set permutation and Signal2Noise ranking metric (Subramanian et al., 2005). After conducting an in-depth literature search, a defined set of Zn homeostasis genes (involved in the concept of nutritional immunity) was included into the analysis (Table S2). The microarray data are deposited at the National Center for Biotechnology Information Gene Expression Omnibus (GSE134016) and are freely available.

Macrophage Cultivation and Infection

Primary bone marrow-derived macrophages (BMDMs) were cultivated as previously described (Bourgeois et al., 2009; Riedelberger et al., 2020). Bone marrow cells from femurs and tibias of C57BL/6 mice (male and female) were cultured in 10 cm square Petri dishes (Thermo Fisher Scientific) at 37 °C, 5 % CO_2 in 9 ml BMDM medium consisting of DMEM (#11584486, Thermo Fisher Scientific), 10 % heat-inactivated fetal calf serum (hiFCS; #F7524, Sigma-Aldrich), 100 µg/ml Penicillin/Streptomycin (Sigma-Aldrich) and 15 % L929 fibroblast cell supernatant. After three days, 5 ml BMDM medium was added and at day 7 of cultivation, the old medium was aspirated and BMDMs were splitted 1:2 in 12 ml fresh BMDM medium. On day 10 of cultivation, adherent cells were harvested by gently scraping with a natural-rubber scraper (Deutsch & Neumann) and pelleted at 300 g, 20 °C for 6.5 min. After counting BMDMs with a CASY counter, BMDMs were seeded in the respective tissue culture plates in BMDM medium and incubated overnight at 37 °C, 5 % CO_2. The experiment was performed on day 11 of cultivation.

Splenic macrophages (SpMs) were cultivated as previously described (Alatery and Basta, 2008). Spleens from male and female WT and *Ifnar1*$^{-/-}$ mice were passed through a 70 µm cell strainer (Corning) and red blood cells (RBCs) were lysed in 2 ml RBC lysis buffer (0.01 M Tris-HCl buffer pH = 7.0 containing 8.3 g/l NH_4Cl; all Sigma-Aldrich) per every spleen. Subsequently, obtained splenocytes were cultured in 12 SpM medium consisting of RPMI-1640 medium (Thermo Fisher Scientific) with 10 % heat-inactivated FCS and 100 µg/ml Penicillin/Streptomycin (both from Sigma-Aldrich) in 10 cm square Petri dishes (Thermo Fisher Scientific). On day 3, non-adherent cells were washed away with 1x PBS and 12 ml fresh SpM medium was replenished. On day 7 of cultivation, adherent SpMs were gently scraped with a natural-rubber scraper and seeded in respective well plates in SpM medium for the following experiment on day 8 of cultivation.

The *Mt1*$^{-/-}$ *Mt2*$^{-/-}$ RAW 264.7 macrophage cell line (clone #3 and #4) (Wu et al., 2017) was cultivated in DMEM (Thermo Fisher Scientific), 10 % hiFCS (Sigma-Aldrich) and 100 µg/ml Penicillin/Streptomycin (Sigma-Aldrich) at 37 °C, 5 % CO_2.

17 h before the infection, BMDMs and SpMs were left untreated or treated with 500 U/ml IFNα or IFNβ (both BioLegend). Where indicated, BMDMs were pre-treated with 10 µM Filgotinib (GLPG0634) (Selleckchem) 60 min before IFNβ treatment. 10 µM freshly-dissolved TPEN, 3 µM Bafilomycin A_1 or 3 mM DPI (all Sigma-Aldrich) was added 60 min before *C. glabrata* infection.

Immune cells were challenged with logarithmic growing fungal strains at a MOI = 2 (multiplicity of infection; 2 yeast cells per immune cell) if not otherwise stated. Throughout *C. glabrata* infection, IFNs-I/Filgotinib/TPEN/Bafilomycin A_1/DPI were left within the cell culture medium.

In Vitro C. glabrata Survival Assays

$1x10^5$ BMDMs were seeded into 96-well plates (at least 4 technical replicates per condition) in 100 µl BMDM medium the day before use. BMDMs were infected with *C. glabrata* (MOI = 0.1; in 50 µl BMDM medium) for 24 h. After BMDM lysis with SDS and scraping, dilutions of cell lysates were plated on YPD plates and CFUs were counted after 48 h at 30 °C.

RNA Isolation and RT-qPCR

$1x10^6$ BMDMs were lysed in 1 ml TRI Reagent® (Molecular Research Center) and RNA was isolated exactly as previously described (Schmittgen and Livak, 2008). After treatment with DNase I (Thermo Fisher Scientific), cDNA was generated using the Reverse Transcription System (Promega) and RT-qPCR was performed by using the Luna® Universal qPCR Master Mix (New England Biolabs) according to manufacturer's instructions. RT-qPCR results from mammalian cells were normalized to *Actb* expression and for fungal gene expression, results were normalized to *ACT1* expression. For RT-qPCR primer sequences, see Table S3.

Immunoblotting

Western blots were performed as describe before (Bourgeois et al., 2009; Riedelberger et al., 2020), whereby $1x10^6$ BMDMs were lysed in 50 µl of 4 % SDS sample buffer (Laemmli, 1970) and incubated at 95 °C for 5 min. Protein samples were loaded onto 10 % SDS-PAGE gels and subsequently transferred to 0.45 µm PVDF membranes (Millipore). After membrane blocking, immunoblotting was conducted overnight at 4 °C with 1:1000 anti-phospho-p40 (#4311), 1:1000 anti-β-Actin (D6A8) (#8457) primary antibodies and by membrane incubation with 1:2000 goat anti-rabbit IgG (H+L) HRP-linked secondary antibody (#7074) (all Cell Signaling). The membranes were incubated in SuperSignal West Pico Chemiluminescent Substrate and were exposed to CL-XPosure films (both Thermo Fisher Scientific).

Quantification of Intracellular Zn Levels

The same setup was used for BMDMs and *ex vivo* SpMs. $2x10^5$ BMDMs were cultivated in 24-well plates and challenged with the respective stimuli. At the end of BMDM challenge after 8 h (unless otherwise stated), BMDMs were washed 3x with PBS and stained with 300 µl of 10 µM Zinpyr (sc-213182, Santa Cruz) in PBS for 30 min at 37 °C. After three PBS washing steps, cells were harvested by trypsinization and resuspended in 300 µl FACS buffer (PBS + 0.1 % BSA). The MFI(Zinpyr) (median fluorescence intensity) of BMDMs was immediately collected on a LSRFortessa (BD Biosciences) and analyzed using FlowJo software version 7.6.5 (FlowJo).

In case of splenic macrophages *in vivo*, WT and *Ifnar1*$^{-/-}$ mice (uninfected or intravenously infected with *C. glabrata*) were sacrificed, spleens were harvested and dissociated via passage through a 70 µM cell strainer. After red blood cell lysis, aliquots of $1x10^6$ splenocytes were transferred

into a 1.5 ml reaction tubes and harvested at 400 g, 4 °C for 5 min. After removing the supernatant, splenocytes were incubated in 50 µl FACS buffer containing 10 µg/ml of anti-CD16/CD32 antibody (BioLegend) for 10 min on ice. Subsequently, 50 µl FACS buffer containing CD11b-APC-Cy7 (#101226), F4/80-PE (#123110) and Ly6C-APC (#128016) (all from BioLegend) were added and splenocytes were stained for 30 min on ice and harvested at 400 g, 4 °C for 5 min. The supernatant was removed and splenocytes were stained in 200 µl 0.1 µM Zinpyr (Santa Cruz) in PBS in a thermomixer for 30 min at 37 °C. After cell harvest, splenocytes were resuspended in 300 µl FACS buffer and immediately analyzed on a LSRFortessa (BD Biosciences) as described above.

Confocal Microscopy

$1x10^5$ BMDMs were prepared and cultured in 200 µl BMDM medium on a 8-well glass bottom µ-slide (ibidi, #80827) the day before the experiment. BMDMs were infected with $mCherry^+$ *C. glabrata* or Calcofluor White-stained wild-type *C. glabrata* (see above) at a MOI = 2. After 4 h, BMDMs were 2x washed with PBS and stained with 200 µl of 10 µM Zinpyr (Santa Cruz) in DMEM for 30 min at 37 °C and, after two PBS washing steps, with 200 µl of 1 µg/ml DAPI in DMEM for 5 min at 37 °C. After three PBS washing steps, cells were cultured in PBS and images were acquired immediately on a Zeiss LSM700 inverse confocal microscope with 40x or 63x plan-apochromat objectives (ZEISS) and visualized using ZEN 2012 software. For lysosomal staining, BMDMs were incubated in 1:500 CytoPainter LysoBlue Indicator Reagent (ab176825, Abcam) in DMEM two hours before the infection. For metallothionein localization in macrophages, *Cg*-infected BMDMs were fixed in 4 % Formaldehyde (Sigma-Aldrich)/PBS for 10 min at room temperature, 3x washed with PBS and permeablized in 0.5 % Tween-20 (Sigma-Aldrich)/PBS for 10 min at room temperature. After three PBS washing steps, cells were incubated in 1 % BSA/0.5 % Tween-20/PBS for 30 min at room temperature and stained with 1:100 anti-metallothionein (UC1MT) primary antibody (ADI-SPA-550-D; Enzo Life Sciences) overnight at 4 °C. Specimens were 3x washed with PBS and incubated with 1:100 goat anti-Mouse IgG (H+L) Alexa Fluor 488-conjugated secondary antibody (A-11001; Invitrogen) for 1 h at room temperature. After staining in 5 µg/ml DAPI for 5 min, the cover slips were washed 3x in PBS before mounting on glass slides with Fluorescence Mounting Medium (S3023, Dako). The slides were dried in the dark at 4 °C and analysis was performed with a Zeiss LSM700 confocal microscope using ZEN 2012 software.

Zinpyr-Based Fungal Zn Acquisition

$1x10^6$ BMDMs were cultivated in 6-well plates and 1000 µl BMDM medium and infected with *C. glabrata* strains (MOI = 1) for 12 h, unless otherwise stated. After 2 h of infection, non-phagocytosed yeast cells were washed away with 2x 2 ml PBS and fresh BMDM medium was added. At the end of infection, BMDMs were washed 3x with 2 ml PBS to remove extracellular fungal cells and BMDMs were lysed with 1 ml 0.005 % ultra-pure SDS (Sigma-Aldrich) in PBS for 15 min on ice. Lysates were harvested by pipetting and fungal cells were pelleted at 21 000 g, 4 °C for 10 min. After completely removing the supernatant, fungal pellet was stained in 10 µl of 500 µM Zinpyr (Santa Cruz) in PBS in a thermomixer for 2 h at 30 °C and 900 rpm. Cells were resuspended in 200 µl PBS and subjected for data collection on a LSRFortessa (BD Biosciences) and analysis by using FlowJo software version

7.6.5 (FlowJo). Fungal cells could be identified by FSC/SSC discrimination, doublet exclusion and, were possible, mCherry expression. The mean fluorescence intensity of Zinpyr was recorded.

Fungal Zn Intoxication Assays

$2x10^5$ BMDMs were infected with *C. glabrata* and at the end of infection, stained with 10 µM Zinpyr (Santa Cruz) for 30 min at 37 °C. After 3x PBS washing steps, BMDMs were lysed with 0.005 % ultra-pure SDS (Sigma-Aldrich) in PBS for 15 min on ice. Fungal cells were pelleted at 21,000 g for 10 min at 4 °C and stained with 2 µg/ml PI (Sigma-Aldrich) in PBS for 5 min at 20 °C. Fungal cells were 1x washed with PBS and resuspended in FACS buffer. Data were immediately collected on a LSRFortessa (BD Biosciences) and analyzed using FlowJo software version 7.6.5 (FlowJo).

Macrophage Cell Sorting

For quantification of the *C. glabrata* survival ratio, BMDMs were infected with $mCherry^+$ *C. glabrata* and after 4 h, stained with 10 µM Zinpyr (Santa Cruz) for 30 min at 37 °C. BMDMs were harvested by trypsinization and immediately purified via FSC/SSC discrimination on a BD FACSAria™ III cell sorter in order to exclude extracellular *C. glabrata*. After SDS lysis of sorted BMDMs, cell lysates were plated on YPD plates and CFUs were counted after 2 days at 30 °C. The *C. glabrata* survival ratio in BMDMs is calculated as the surviving *C. glabrata* CFUs per sorted BMDMs divided by the total amount of *C. glabrata* per sorted BMDM, represented by mCherry fluorescence (median fluorescence intensity).

For RNA isolation of intracellular *C. glabrata*, at the end of the infection, BMDMs were harvested by trypsinization and immediately purified via FSC/SSC discrimination on a BD FACSAria™ III cell sorter. Sorted BMDMs were lysed in 1 ml TRI Reagent® (Molecular Research Center) and fungal RNA was isolated as described previously (Tscherner et al., 2012).

Intracellular ROS Assays

Immediately before macrophage challenge, $2x10^5$ BMDMs were washed 3x with PBS, cultivated in 280 µl DMEM and infected with *C. glabrata* (MOI = 10; in 20 µl PBS) for 2 h. After 1.5 h, 20 µl of 170 µM DHE (f.c. 10 µM) (Santa Cruz) was added to the BMDM medium and incubated for 30 min. Subsequently, BMDMs were washed 3x with PBS, harvested by trypsinization and resuspended in 300 µl FACS buffer. The MFI(DHE) (median fluorescence intensity) of BMDMs was collected on a LSRFortessa (BD Biosciences) and analyzed using FlowJo software version 7.6.5 (FlowJo).

Sample Preparation and Total Metal Quantification by ICP-MS

$1x10^6$ BMDMs were cultivated in 6-well plates and 1000 µl BMDM medium and infected with *C. glabrata* (MOI = 2) for 8 h. At the end of infection, BMDMs were washed 2x with 2 ml PBS, and excessive liquids were removed. After adding 100 µl 0.1 % ultra-pure SDS (Sigma-Aldrich) in HPLC water to the respective wells, BMDMs were scraped and lysates were transferred into 1.5 ml reaction tubes and incubated on ice for 20 min. Samples were sonicated in a Bioruptor (Diagenode), centrifuged at 25 000 g, 4 °C for 15 min, and lysates were transferred into new 1.5 ml reaction tubes and stored at -80 °C.

50 µl BMDM lysates were digested in closed PFA vials with 200 µL nitric acid (65 %, p.a. grade, Emsure) and 100 µL of hydrogen peroxide (30 %, supra-pure quality, Merck) at 80 °C for 2-3 hours. The solutions were diluted to a final volume of 5 mL with 1 % (v/v) nitric acid. Standard solutions with defined concentrations of Mg, Ca, Mn, Fe, Ni, Cu and Zn were prepared by appropriate dilution of CertiPUR multi-element standard VII (100 mg/l, Merck). Indium was used as internal standard for the ICP-MS measurements and was, therefore, spiked to all samples and standard solutions at a final concentration of 10 µg/l.

Quantitative analysis of the target analytes was accomplished using inductively coupled plasma-mass spectrometry (ICP-MS), an analytical technique which allows multi-element investigations even at extremely low concentration levels. Measurements were performed using an iCAP Qc quadrupole ICP-MS instrument (Thermo Fisher Scientific) equipped with a standard quartz tube torch and nickel sample and skimmer cones. Sample and standard solutions were brought into the ICP by means of a pneumatic nebulizer (concentric, material: Teflon) and a cyclonic glass spray chamber. Sample uptake was performed by the peristaltic pump of the iCAP Qc via a SC2-DX autosampler (Elemental Scientific) in combination with a FAST sample introduction system (1 mL sample loop, Elemental Scientific). ICP-MS analysis was carried out in "KED" mode (kinetic energy discrimination) for the separation of spectral interferences caused from polyatomic ions produced in the argon plasma. A mixture of 7 % hydrogen in helium was used as collision gas at a flow rate of 5 ml/min. A dwell time of 10 ms and 50 sweeps per reading and 4 replicates per sample was set. For analysis, the isotopes ^{25}Mg, ^{43}Ca, ^{55}Mn, ^{57}Fe, ^{60}Ni, ^{63}Cu, ^{66}Zn and ^{115}In were monitored by scanning the appropriate m/z ratios. Prior to measurement the ICP-MS instrument settings were optimized using a tune solution containing 1 µg/l of indium, barium, uranium and cerium to achieve satisfying sensitivity and oxide ratios ($CeO^+/Ce^+ < 2$ %). Data acquisition was performed using Qtegra software provided by the manufacturer of the instrument. Internal standardization with indium was carried out to compensate for potential instrument instability and signal drift. An external calibration function, using aqueous standard solutions, was used for quantification of the derived analyte signal.

Data Analysis and Statistics

Data are represented as mean ± SD. Statistical analysis was performed with GraphPad Prism version 5.04 for Windows (GraphPad). If not otherwise stated, differences between two mean values were evaluated by using unpaired t-tests with 95 % confidence intervals. Multiple groups were compared by one-way ANOVA with Bonferroni’s post hoc analysis. (* p-value < 0.05; ** p-value < 0.01; *** p-value < 0.001; ns, not statistically significant)

Supplemental References

Alatery, A., and Basta, S. (2008). An efficient culture method for generating large quantities of mature mouse splenic macrophages. J. Immunol. Methods *338*, 47–57.

Alker, W., and Haase, H. (2018). Zinc and sepsis. Nutrients *10*, 1–17.

Alonzi, T., Maritano, D., Gorgoni, B., Rizzuto, G., Libert, C., and Poli, V. (2002). Essential Role of STAT3 in the Control of the Acute-Phase Response as Revealed by Inducible Gene Activation in the Liver. Mol. Cell. Biol. *21*, 1621–1632.

Boer, J. De, Williams, A., Skavdis, G., Harker, N., Coles, M., Norton, T., Williams, K., Roderick, K., and Potocnik, A.J. (2003). Transgenic mice with hematopoietic and lymphoid specific expression of Cre. Eur. J. Immunol. *33*, 314–325.

Bonaventura, P., Benedetti, G., Albarède, F., and Miossec, P. (2015). Zinc and its role in immunity and inflammation. Autoimmun. Rev. *14*, 277–285.

Bourgeois, C., Majer, O., Frohner, I., and Kuchler, K. (2009). *In vitro* systems for studying the interaction of fungal pathogens with primary cells from the mammalian innate immune system. Methods Mol. Biol. *470*, 125–139.

Catalanotti, F., Reyes, G., Jesenberger, V., Galabova-Kovacs, G., De Matos Simoes, R., Carugo, O., and Baccarini, M. (2009). A Mek1-Mek2 heterodimer determines the strength and duration of the Erk signal. Nat. Struct. Mol. Biol. *16*, 294–303.

Clausen, B.E., Burkhardt, C., Reith, W., Renkawitz, R., and Forster, I. (1999). Conditional gene targeting in macrophages and granulocytes using LysMcre mice. Transgenic Res. *8*, 265–277.

Cui, Y., Riedlinger, G., Miyoshi, K., Tang, W., Li, C., Deng, C.-X., Robinson, G.W., and Hennighausen, L. (2004). Inactivation of *Stat5* in Mouse Mammary Epithelium during Pregnancy Reveals Distinct Functions in Cell Proliferation, Survival, and Differentiation. Mol. Cell. Biol. *24*, 8037–8047.

Durbin, J.E., Hackenmiller, R., Simon, M.C., and Levy, D.E. (1996). Targeted disruption of the mouse *Stat1* gene results in compromised innate immunity to viral disease. Cell *84*, 443–450.

Gammoh, N.Z., and Rink, L. (2017). Zinc in infection and inflammation. Nutrients *9*.

Gao, H., Dai, W., Zhao, L., Min, J., and Wang, F. (2018). The Role of Zinc and Zinc Homeostasis in Macrophage Function. J. Immunol. Res. *2018*, 1–11.

Gentleman, R.C., Carey, V.J., Bates, D.M., Bolstad, B.M., Dettling, M., Dudoit, S., Ellis, B., Gautier, L., Ge, Y., Gentry, J., et al. (2004). Bioconductor: open software development for computational biology and bioinformatics. Genome Biol *5*, R80.

Gibson, D.G., Young, L., Chuang, R.-Y., Venter, J.C., Hutchison, C.A. 3rd, and Smith, H.O. (2009). Enzymatic assembly of DNA molecules up to several hundred kilobases. Nat. Methods *6*, 343–345.

Günther, V., Lindert, U., and Schaffner, W. (2012). The taste of heavy metals: Gene regulation by MTF-1. Biochim Biophys Acta *1823*, 1416–1425.

Hayashi, S., Lewis, P., Pevny, L., and Mcmahon, A.P. (2002). Efficient gene modulation in mouse epiblast using a Sox2Cre transgenic mouse strain. Gene Expr Patterns *2*, 93–97.

Kaiser, C., Michaelis, S., and Mitchell, A. (1994). Methods in Yeast Genetics. A Laboratory Course Manual (New York: Cold Spring Harbor Laboratory Press).

Kaplan, M.H., Schindler, U., Smiley, S.T., and Grusby, M.J. (1996). Stat6 is required for mediating responses to IL-4 and for the development of Th2 cells. Immunity *4*, 313–319.

Kauffmann, A., Gentleman, R., and Huber, W. (2009). arrayQualityMetrics - A bioconductor package for quality assessment of microarray data. Bioinformatics *25*, 415–416.

Kimura, T., Kadokawa, Y., Harada, H., Matsumoto, M., Sato, M., Kashiwazaki, Y., Tarutani, M., Tan, R.S., Takasugi, T., Matsuyama, T., et al. (1996). Essential and non-redundant roles of p48 (ISGF3 gamma) and IRF-1 in both type I and type II interferon responses, as revealed by gene targeting studies. Genes Cells *1*, 115–124.

Krauke, Y., and Sychrova, H. (2011). Cnh1 Na+/H+ antiporter and Ena1 Na+-ATPase play different roles in cation homeostasis and cell physiology of *Candida glabrata*. FEMS Yeast Res. *11*, 29–41.

Laemmli, U.K. (1970). Cleavage of structural proteins during the assembly of the head of bacteriophage T4. Nature *227*, 680–685.

Lopez, C.A., and Skaar, E.P. (2018). The Impact of Dietary Transition Metals on Host-Bacterial Interactions. Cell Host Microbe *23*, 737–748.

Luo, J., Zhang, L., Dorfman, A.L., Sherwood, M.C., Logsdon, M.N., Horner, J.W., Depinho, R.A., Izumo, S., and Cantley, L.C. (2005). Class IA Phosphoinositide 3-Kinase Regulates Heart Size and Physiological Cardiac Hypertrophy. Mol. Cell. Biol. *25*, 9491–9502.

Masters, B., Kelly, E., Quaife, C., Brinster, R., and Palmiter, R. (1994). Targeted disruption of metallothionein I and II genes

increases sensitivity to cadmium. Proc. Natl. Acad. Sci. *91*, 584–588.

Müller, U., Steinhoff, U., Reis, L.F.L., Hemmi, S., Paviovic, J., Zinkernagel, R.M., and Aguet, M. (1994). Functional Role of Type I and Type II Interferons in Antiviral Defense. Science (80-.). *264*, 1918–1921.

Park, C., Li, S., Cha, E., and Schindler, C. (2000). Immune response in *Stat2* knockout mice. Immunity *13*, 795–804.

Rahman, M.T., and Karim, M.M. (2018). Metallothionein: A potential link in the regulation of zinc in nutritional immunity. Biol. Trace Elem. Res. *182*, 1–13.

Reis, L.F.L., Ruffner, H., Stark, G., Aguet, M., and Weissmann, C. (1994). Mice devoid of interferon regulatory factor 1 (IRF-1) show normal expression of type I interferon genes. EMBO J. *13*, 4798–4806.

Reuss, O., Vik, Å., Kolter, R., and Morschhäuser, J. (2004). The SAT1 flipper, an optimized tool for gene disruption in *Candida albicans*. Gene *341*, 119–127.

Rice, J.M., Zweifach, A., and Lynes, M.A. (2016). Metallothionein regulates intracellular zinc signaling during CD4+T cell activation. BMC Immunol. *17*, 13.

Riedelberger, M., Penninger, P., Tscherner, M., Strobl, B., Weiss, G., Kuchler, K., Riedelberger, M., Penninger, P., Tscherner, M., Seifert, M., et al. (2020). Type I Interferon Response Dysregulates Host Iron Homeostasis and Enhances *Candida glabrata* Infection. Cell Host Microbe *27*, 454-466.e8.

Sapkota, M., and Knoell, D. (2018). Essential Role of Zinc and Zinc Transporters in Myeloid Cell Function and Host Defense against Infection. J Immunol Res *2018*.

Sato, M., Suemori, H., Hata, N., Asagiri, M., Ogasawara, K., Nakao, K., Nakaya, T., Katsuki, M., Noguchi, S., Tanaka, N., et al. (2000). Distinct and Essential Roles of Transcription Factors IRF-3 and IRF-7 in Response to Viruses for IFN-alpha/beta Gene Induction. Immunity *13*, 539–548.

Schmittgen, T.D., and Livak, K.J. (2008). Analyzing real-time PCR data by the comparative CT method. Nat. Protoc. *3*, 1101–1108.

Sheldon, J.R., and Skaar, E.P. (2019). Metals as phagocyte antimicrobial effectors. Curr. Opin. Immunol. *60*, 1–9.

Shimoda, K., Kato, K., Aoki, K., Matsuda, T., Miyamoto, A., Shibamori, M., Yamashita, M., Numata, A., Takase, K., Kobayashi, S., et al. (2000). Tyk2 plays a restricted role in IFNα signaling, although it is required for IL-12-mediated T cell function. Immunity *13*, 561–571.

Smyth, G.K. (2004). Linear models and empirical bayes methods for assessing differential expression in microarray experiments. Stat. Appl. Genet. Mol. Biol. *3*, Article3.

Subramanian, A., Tamayo, P., Mootha, V.K., Mukherjee, S., Ebert, B.L., Gillette, M.A., Paulovich, A., Pomeroy, S.L., Golub, T.R., Lander, E.S., et al. (2005). Gene set enrichment analysis: A knowledge-based approach for interpreting genome-wide expression profiles. Proc. Natl. Acad. Sci. *102*, 15545–15550.

Subramanian Vignesh, K., and Deepe, G.J. (2017). Metallothioneins: Emerging modulators in immunity and infection. Int. J. Mol. Sci. *18*.

Subramanian Vignesh, K., and Deepe, G.S. (2016). Immunological orchestration of zinc homeostasis: The battle between host mechanisms and pathogen defenses. Arch. Biochem. Biophys. *611*, 66–78.

Suzuki, A., Yamaguchi, M., Ohteki, T., Sasaki, T., Kaisho, T., Kimura, Y., Yoshida, R., Wakeham, A., Higuchi, T., Fukumoto, M., et al. (2002). T Cell-Specific Loss of Pten Leads to Defects in Central and Peripheral Tolerance. Immunity *14*, 523–534.

Tscherner, M., Stappler, E., Hnisz, D., and Kuchler, K. (2012). The histone acetyltransferase Hat1 facilitates DNA damage repair and morphogenesis in *Candida albicans*. Mol. Microbiol. *86*, 1197–1214.

Tscherner, M., Zwolanek, F., Jenull, S., Sedlazeck, F.J., Petryshyn, A., Frohner, I.E., Mavrianos, J., Chauhan, N., von Haeseler, A., and Kuchler, K. (2015). The *Candida albicans* Histone Acetyltransferase Hat1 Regulates Stress Resistance and Virulence via Distinct Chromatin Assembly Pathways. PLoS Pathog. *11*, e1005218.

Wu, A., Tymoszuk, P., Haschka, D., Heeke, S., Dichtl, S., Petzer, V., Seifert, M., Hilbe, R., Sopper, S., Talasz, H., et al. (2017). *Salmonella* Utilizes Zinc To Subvert Antimicrobial Host Defense of Macrophages via Modulation of NF-κB Signaling. Infect Immun *85*, e00418-17.

Yáñez-Carrillo, P., Orta-Zavalza, E., Gutiérrez-Escobedo, G., Patrón-Soberano, A., De Las Peñas, A., and Castaño, I. (2015). Expression vectors for C-terminal fusions with fluorescent proteins and epitope tags in *Candida glabrata*. Fungal Genet. Biol. *80*, 43–52.
